# Insights into the Development of Corrosion Protection Coatings

**DOI:** 10.3390/polym17111548

**Published:** 2025-06-02

**Authors:** Monmi Saikia, Trisha Dutta, Niteen Jadhav, Deep J. Kalita

**Affiliations:** 1 Department of Chemistry, Eastern Karbi Anglong College, Karbi Anglong 782480, Assam, India; monmi.saikia@gmail.com (M.S.); trishabborooah@gmail.com (T.D.); 2 ChemCo Systems, 2800 Bay Rd, Redwood City, CA 94063, USA; n3.uict@gmail.com; 3 Elinor Coatings LLC, 1805 NDSU Research Park Dr N, Fargo, ND 58102, USA

**Keywords:** corrosion, polymer coatings, nanocomposite, epoxy resin

## Abstract

This review article focuses on providing an accumulated knowledge on state-of-the-art composite polymer coating technologies that are studied for corrosion protection. A specific focus has been given to epoxy resin-based composite systems, considering their wide use due to remarkable chemical resistance, excellent adhesion to substrate, thermal stability, and mechanical strength. The addition of various functional polymers to the epoxy matrix has spurred significant advancements in the prevention of corrosion. Light has been shed on the epoxy resin composite systems that are produced by blending with functional polymers like conductive polymers, hydrophobic polymers, etc., and nanofillers. In many cases, the formation of a passive layer at the metal/polymer interface was aided by the addition of such a functional polymer and nanofiller to the epoxy matrix. As a result, corrosive ions are prevented from penetrating by the physical barrier that composite coatings provide. Comparable blends of epoxy and polyamide, epoxy and polyester, and epoxy/poly(vinyl alcohol) and epoxy/polyurethane have superior adhesion, wear, barrier, and anticorrosion properties due to the fine dispersion of nanocarbon and inorganic nanoparticles. The several strategies used to prevent metals from corroding are covered in this review article.

## 1. Introduction

### 1.1. Corrosion

“Corrosion” is the deterioration of a material caused by the action of either the environment alone or in alliance with the mechanical forces. In the case of metals, corrosion generally refers to oxidation, which is an electrochemical phenomenon. However, it also includes the various kind of interactions of a metal and alloy (solid or liquid) with its surrounding environment regardless of whether the interaction is beneficial or adverse. It is the tendency of the material to be in its lowest energy state that forms the background behind the phenomenon of metallic corrosion. The term “corrosion” also considers some other cases such as the deterioration of stone, wood, or plastic, where there is no involvement of any electrochemical processes [1,2]. Corrosion initiates loss, which touches the global infrastructure, causing major effects in its economic and ecological aspects [3,4,5]. Owing to the enormous variety of corrosive environments and diversities of corrosion reactions involved, the classification of corrosion becomes a complicated task. However, there are broad ways of organizing the different corrosions by labelling them under separate levels.

### 1.2. Classification of Corrosion

One of the routes for classifying corrosion is by categorizing it two different modes: (a) intrinsic mode and (b) extrinsic mode. When corrosion takes place independent of the materials design, such as by pitting, parting, stress corrosion cracking, and intergranular corrosion, it comes under “intrinsic mode”. However, when corrosion is affected by the design of the material, it comes under the “extrinsic mode”. This mode of corrosion comes under the crevice or deposit corrosion, erosion corrosion [6,7]. Another broad classification of corrosion brings ‘wet’ and ‘dry’ corrosion reactions under it [1]. Along with humidity, air, and various types of water (freshwater, seawater, and distilled water) acting as the causes for corrosion, there are also other factors accompanying this environment, such as hydrogen sulfide, fuel gases, ammonia, etc. [8,9]. A metal becomes affected by corrosion in several ways, which is based on the nature and the precise conditions of the environment prevailing, which gives rise to a broad classification of the various forms of corrosion containing a total of five types. These are as follows:

(a) Uniform (or almost uniform), in which there is corrosion of all areas of the metal at the same (or a similar) rate, for example, oxidation and tarnishing.
(b) Localized corrosion, where there is higher rate of corrosion in some areas of the metal surface because of the presence of ‘heterogeneities’ in the metal, the environment, or the geometry of the structure as a whole. Examples include crevice corrosion, filiform corrosion, and deposit attack.
(c) Pitting, where there is a highly localized attack at specific areas causing the formation of small pits that penetrate the metal and might lead to perforation, for example, pitting of passive metals such as aluminium alloys, etc.
(d) Selective dissolution, where in an alloy, usually, the most active component is selectively removed from an alloy. Examples include, de-aluminification, graphitization, etc.
(e) Conjoint action, where corrosion and a mechanical factor act together, for example, a localized attack or fracture due to the combined action of a mechanical factor and corrosion [1].

### 1.3. Factors Affecting Corrosion

The major factors that influence the rate of corrosion are primarily the nature of the metal and the nature of the corroding environment. Along with these other factors, some other causes that affect corrosion are the purity of the metal, nature of the surface film, nature of the corrosive product, temperature, humidity of the air, different salts and their levels, and the pH of the electrolyte.

The nature of the metal also relies on its position in the galvanic series, the type of surface film, the impurities of alloying elements in the metal, and the nature of the corrosive products. When there are two metals of different types that are connected electrically in a given electrolyte, the one having higher oxidation potential or the metal that occupies a higher level in the galvanic series becomes corroded, thereby protecting the rest. The difference in the positions of the two metals in the galvanic series also affects the corrosion rate. The greater the difference in the position of the two metals i.e., the higher the difference is, the faster the rate of corrosion.

The rate of corrosion is proportional to the addition of impurities in the metal. This is caused due to the formation of tiny electrochemical cells where the anodic part becomes corroded. For example, impurities like Fe, or Pb present in Zn metal, increases the rate of corrosion.

The metals have a tendency to be covered with a thin surface film of the metal oxide in an aerated atmosphere. “Specific volume ratio” reveals the ratio of volumes of the metal oxide to the metal, which, in turn, determines the effect of the surface film. The higher the ratio, the less the oxidation rate.

If the products formed in the corrosion are found to be soluble in the corrosive medium, then the corrosion occurs at a faster rate. Also, corrosion is further exceeded when the corrosive product formed is volatile, which, when it evaporates as soon as it forms, exposes the metal surface for further attack.

The nature of the corroding environment further depends on the temperature, humidity of the air, and effect of pH. The corrosion rate increases with the increase in temperature. For every 10 °C rise in temperature, it is expected that there will be a double rate of increase in corrosion, provided other biological conditions are kept constant. The humidity of the air also plays a decisive role in the rate of corrosion. Above a special point of relative humidity, the rate of corrosion increases, which is called the critical humidity. This is because the oxide film has a tendency of absorbing moisture, thereby creating another electrochemical corrosion and, hence, enhancing the corrosion rate.

The pH is one of the most important determining factors for the corrosion rate. Corrosion is higher when the pH is lower. Corrosive media that are acidic in nature with a pH less than 7 are more susceptible to corrosion than alkaline or neutral media [10]. Potential-pH diagrams also known as Pourbaix diagrams for different metals indicate the regions of corrosion, passivation, and immunity observed for a given metal under different potential and pH conditions. Pourbaix diagrams indicate the thermodynamic propensity of corrosion at a given pH.

Anti-corrosive functional polymeric coatings have received significant attention and flexibility against corrosion protection as they can be tuned with respect to their design and formulation to achieve more than a single protective mechanism as compared to the non-polymeric ones. This review, thus, sheds light on the recent investigations on the synthesis of anti-corrosive nanostructured polymeric coatings for preventing corrosion in an impactful manner.

The rate of corrosion mainly depends on two factors:
**Nature of metal****Nature of corroding environment**This further depends upon the following:This further depends upon the following:(a) Position in galvanic series;(a) Temperature;(b) Purity of metal;(b) Humidity of air;(c) Nature of surface film;(c) Effect of pH.(d) Nature of corrosive product.


## 2. Corrosion Control by Use of Coatings

Corrosion has been one of the most consequential problems in our society that results in huge losses each year amounting to hundreds of billions of dollars [11]. Some other losses caused due to corrosion include the damage of industrial machines and metallic equipment, such as boilers, and even unpredictable machinery failure that would lead to loss of life. It thereby reduces the overall value of the product and causes the wastage of unpredictable machinery failure and down time. Due to corrosion, some metallic properties such as conductivity, ductility, malleability, luster, etc., are also lost [10]. Thus, it becomes a challenge to check this economic loss, which is estimated to be about 3.4% of the world’s GDP [12].

In the metallurgical and electronic manufacturing industries, corrosion control is a major subject of increasing importance. Among the most important problems facing the world today is metal corrosion. Paint, or an organic layer applied to the metal substrate is the most often used corrosion prevention method. Better yet, longer-lasting corrosion resistance can be achieved by using materials coated organically. Furthermore, this benefit depends on the coating’s ability to shield the coated metal layer against defects that may arise throughout its useful life [13]. The method that is most frequently employed can be polymer coatings [14]. Also, because conducting polymers have strong anti-corrosion capabilities and are environmentally friendly, they are becoming more and more popular as film-developing corrosion protective coatings.

### 2.1. Coatings

**Figure 1:**
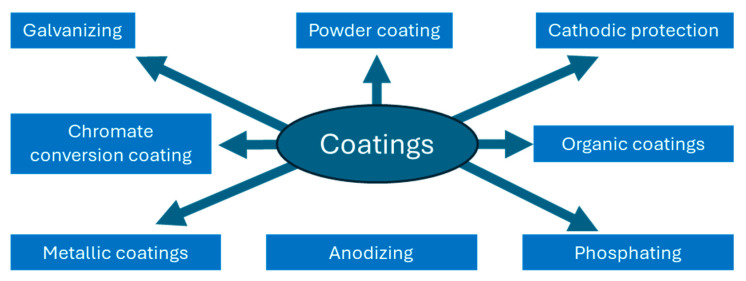
Coatings for corrosion mitigation.

Examples of common techniques employed by paint/coatings for the corrosion management of steel substrates are discussed here, notwithstanding the nearly infinite variations in pre-treatment and coating development. A few examples are galvanizing, powder coating, chromate conversion coating, ceramic coatings, epoxy coatings, metallic coatings, organic coatings, phosphating, anodizing, paints and primers, and vinyl coatings. Paints and coatings are necessary to stop steel from corroding, and ordinary paint content must be replaced with harmless, ecologically acceptable compositions (Figure 1).

### 2.2. Polymer Coatings

A polymer coating is a paint or thin layer of coating consisting of polymers that offer excellent adhesion and corrosion resistance. Polymer coatings are omnipresent in today’s world. They serve specific purposes and are utilized for protection and ornamentation. Polymer coatings are generally classified as superior coating systems due to their low cost, increased performance, and ease of supply. An excellent family of protective materials, polymer coatings are applied to a wide range of substrates in almost every industry, including construction, electronics, automotive, aircraft, and rail. The primary goal of their development is to shield vital technical components from hostile exterior environments. Typically, a polymer matrix and erratically dispersed micro/nano dielectric particles serve as fillers in polymeric coatings. Currently, the scientific community is paying close attention to several advancements in traditional coating systems that have been introduced by the research community, such as multifunctional smart coating systems. Examples of these advancements include superhydrophobic coatings and self-healing, self-cleaning, and super-hydrophilic coatings. An elastomer or other polymeric material is applied to a supporting substrate through the polymeric coating technique. Polymeric coatings include, for example, natural and synthetic rubber, urethane, polyvinyl chloride, acrylic, epoxy, silicone, phenolic resins, and nitrocellulose. Salts and chemicals also play an important role in corrosion protection by hindering electrochemical reactions that lead to the degradation of metals. The nature of metal, the overall condition of the environment, and the quantity of aggressive ions in the medium all affect how much corrosion occurs. For instance, iron is isolated, and corrosion is subsequently reduced when CO_3_^−2^ and NO_3_^−^ form an insoluble deposit on its surface. Halide ions, on the other hand, selectively adsorb over the metal surface and stop the oxide phase from forming, which leads to ongoing corrosion. Both domestically and industrially, iron, aluminum, and their alloys are frequently used [15]. Salt of nitrate and sulphate have been utilized as inhibitors to prevent corrosion in various corrosive situations [16,17].

### 2.3. Classification of Polymer Coatings

Based on the type of resin used as the primary component, polymer coatings for corrosion protection can be classified. This classification is not exhaustive. In anti-corrosive coatings, epoxy, polyester, polyurethane, and acrylic are the most often used resin types. These are called traditional polymer coatings, widely used for the protection of metal materials. Organic compounds, which are conductive like metals, are called conductive polymers. Conductive polymers offer numerous benefits such as functioning as anticorrosion coatings because of their unique structures and capabilities.

The other types of polymer coatings used for corrosion protection include alkyd resins, a type of synthetic polyester resin, which is used to make protective coatings, paints, varnishes, and enamels. Inorganic resin is a composition made from an aqueous solution of metal phosphate, an oxy-boron compound, a wollastonite compound, and other additives, which are used to make protective coatings. Thermoplastic coating is a thick, resilient, and versatile industrial coating to make a protective layer. Chlorinated rubber resins are used to prepare coatings, inks, and adhesives.

### 2.4. Corrosion Testing Methods

Corrosion testing methods include several techniques for evaluating the corrosion protection ability for each coating system. One of the most frequently used techniques involves the salt spray test, where the coated specimens are exposed to a lofty corrosive salt fog ambience, assessing their resistance to corrosion.

Another extensively employed test is electrochemical impedance spectroscopy (EIS), measuring the impedance response of the specimen to the electrochemical signals, thereby providing significant information regarding the barrier properties of the coating along with its corrosion resistance. Some tests, such as pull-off tests or cross-cut tests, are used to evaluate the strength of the bonding between the coating and the substrate. Other tests include the mechanical tests, which involve abrasion resistance tests, and the hardness tests, which estimate the mechanical properties of the coatings and their potential for standing up to the external stresses. Some techniques also display an escalated assessment of the coatings, thereby expediting the development and optimization of corrosion protection coatings. Evaluation of the performance can also be carried out using cyclic corrosion testing, which is a blend of alternating temperature cycling, alternating wet and dry environments, and subjecting the coating to the corrosive agents for replicating the cyclic nature of corrosion in a compressed time frame. Accelerated weathering testing forms another approach for testing that comprises a blend of temperature fluctuations, UV radiation, and moisture for the purpose of stimulating the effects of prolonged time outdoor exposure. Some additional techniques consist of scanning electron microscopy (SEM) and X-ray diffraction (XRD), useful in the scenario of phase identification and microstructural analysis, which assists in better understanding of the coating’s composition and structure [18].

## 3. Protective Mechanism of Anti-Corrosive Coating

Anti-corrosive coatings protect the metals from corrosion by different mechanisms. These mechanisms cover a variety of tactics, such as self-healing qualities, sacrificial protection, and the barrier effect, each of which uses a unique approach to solve corrosion issues. For the protection of metals from corrosion, various methods are employed, which include the following:

Barrier formation: When water, oxygen, and corrosion ions are the corrosive agents, the protective coating acts like a physical barrier to reduce the rate of ingress of such chemicals contacting the material by acting as a physical barrier.

Disrupting the electrochemical corrosion cell: This involves increasing the electrical resistance at the interface between the material and the electrolyte.

**Corrosion inhibitors:** Corrosion inhibitors can work by increasing the electrical resistance of the metallic surface, by adsorbing onto the metallic surface to form a film, or by reducing the movement of ions to the metallic surface.

**Self-healing properties:** Some coatings that prevent corrosion have a novel self-healing mechanism. These coatings incorporate components or additives that can self-heal when they incur slight scratches or mechanical damage [19].

**Figure 2:**
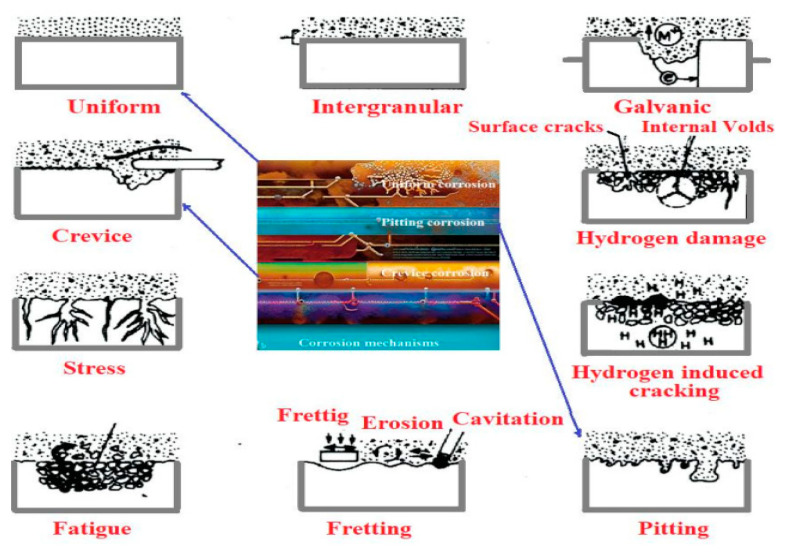
Corrosion mechanisms and protection strategies (adopted from references [18,20]).

These coatings can mend themselves for continuous protection, and that is maintained even in the event of minor flaws or breaches in the coating film. This property has a high valuation because it lessens the possibility that localized flaws may affect overall corrosion resistance. Figure 2 shows the various kinds of corrosion protection mechanisms, such as creative corrosion, uniform corrosion, and pitting corrosion, along with the corresponding corrosion prevention methods offered by corrosion protection coatings [18,20]. It visually depicts how coatings act as a barrier between the corrosive environment and the substrate, preventing the initiation and propagation of corrosion. It shows how coatings prevent corrosion from starting and spreading by acting as a barrier between the substrate and the corrosive environment.

## 4. Development in Nanostructured Anti-Corrosive Polymer Coating

**Figure 3:**
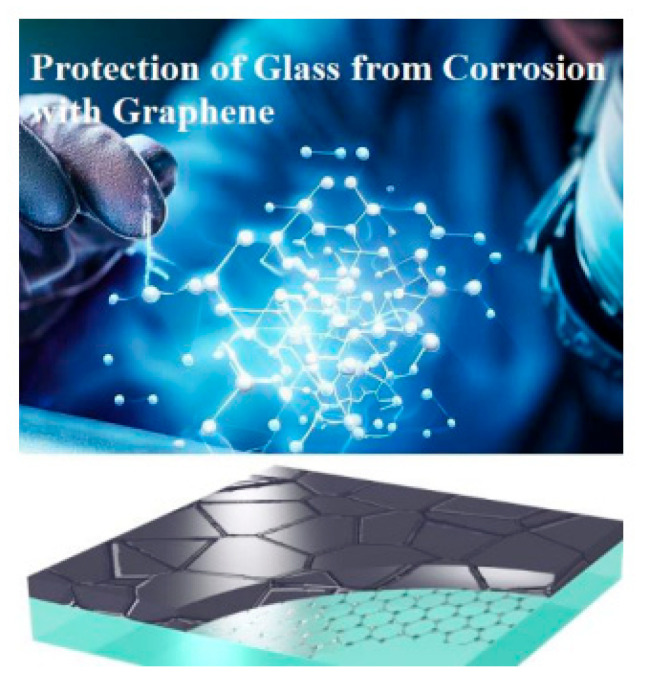
Nanotechnology in corrosion protection coatings (adopted from reference [18]).

One of the most common methods for preventing metal corrosion is to apply anti-corrosion paints to surfaces. Common anti-corrosion paints (e.g., epoxy, polyurethane, polyester, etc.) are made of non-long-lasting thermosetting resin. Once the coating is scratched or a hole is formed, the corrosion can easily attack the matrix. Anticorrosion coatings must contain corrosion inhibitors such as lead compounds and chromosome pigments to improve their corrosion performance [21]. However, the environment protection authority and other environmental organizations require the removal of lead and chromium from usage as pigments in anticorrosion paint due to their harmful effects. Zinc particles are used as pigments in sacrificial coatings, which are intended to provide strong protection while reducing pollution. In the last 20 years, conducting polymers have drawn a lot of attention due to their exceptional ability to prevent metal corrosion and their environmentally beneficial nature [22,23,24,25,26,27,28]. Of these conducting polymers, polyaniline is regarded as one of the most effective anti-corrosion substances [29,30,31]. Additionally, by adding nanoparticles, which improve mechanical qualities, barrier performance, and other special features, the use of nanotechnology has made it possible to improve organic coatings, which is shown in Figure 3 [18,32].

### 4.1. Epoxy-Based Nanocomposite Polymer Coatings

Epoxy resin is a thermosetting polymer with several applications [33,34]. Thermoplastic and conductive polymers have been used to create epoxy blends [35,36]. Blending has been found to improve the chemical and physical properties of epoxy, including high adhesion, high diffusion resistance against corrosive ions, high electrical conductivity, chemical resistance, and stability against temperature changes and mechanical shock. Because of their exceptional adherence to metals, high chemical stability, and resistance to heat and water, epoxy resins have found widespread application in high-performance coatings [37]. They are produced by the condensation of epichlorohydrin and diphenyl propane derivatives in the presence of a basic catalyst. Bisphenol A (BPA) is the diphenyl propane that is most frequently utilized to create solvent-borne epoxy resins. When making epoxy resins, glycerol or other aliphatic polyols can be used instead of bisphenol A. Numerous synthetic routes resulted in the creation of many epoxy resins, including Novolac, which contains F, P, and Si, as well as tetrafunctional, trifunctional, and cycloaliphatic resins and the majority of the more recent bio-based ones. The choice of resin type and curing technique in the coating formulation is largely determined by the ultimate use of the epoxy resin [38].

#### 4.1.1. Epoxy/Polyaniline

By using inorganic nanolayers consisting of clay and camphorsulfonic acid (CSA) and ammonium peroxydisulfate (APS), which play the role of a surfactant and initiator, respectively, in situ emulsion polymerization has been used to successfully prepare a series of polyaniline (PANI)/montmorillonite (MMT) nanocomposite materials. The nanocomposites were bound together using epoxy glue to achieve a thick and consistent coating. Electrochemical impedance spectroscopy (EIS) was used to examine the epoxy I, epoxy/polyaniline (EP), and epoxy blend with polyaniline/MMT (EPM) coatings to determine the impact of MMT and PANI on the performance of corrosion inhibition of the coatings in a saline solution of 3.5% at 65 °C. The findings demonstrated that EPM coatings with 5% clay with alumina showed improved corrosion resistance. The resistivity of epoxy is greatly increased when PACN nanocomposites are incorporated. In an electrochemical impedance spectroscopy study of an anodized aluminum at 65 °C with 3.5 wt.% water NaCl electrolyte, the corrosion protection effect of low-clay-load PACN materials (0.5%, 1%, and 5%) was shown. EIS was employed to study the performance of corrosion protection of the nanocomposites mixed with epoxy [39]. The corrosion protection of epoxy coatings, including polyaniline lignosulfonate-doped polyaniline (Pani-LGS) on Aluminium alloy AA2024-T3, was examined using a 0.6 M NaCl solution. It was observed that the performance of 5 wt.% Pani-LGS/epoxy blend coating showed no corrosion after 30 days of exposure to a 0.6 M NaCl solution. The mechanism of corrosion protection was also described. On AA2024, adherent, corrosion-resistant Pani-LGS coatings were effectively created using the chemical oxidative polymerization of aniline with a lignosulfonic acid dopant. For the first time, the performance of these coatings has been disclosed. Particle dispersibility is low when the Pani-LGS level in the epoxy coating composition is low. On the other hand, because of agglomeration, a high content and large volume proportion of particles reduces coating performance [40]. This article describes the production of an epoI(E) covering that inhibits corrosion on carbon steel grade ST37 by blending an organic–inorganic hybrid nanocomposite [41]. It was found that using an in situ chemical oxidative method of aniline monomers in the presence of ZnO nanorods and camphorsulfonic acid (CSA) and ammonium peroxydisulfate (APS) playing the role of surfactant and initiator, respectively, the preparation of a series of conducting polyaniline (PANI)–ZnO nanocomposites materials has been successfully conducted. Using electrochemical techniques such as electrochemical impedance spectroscopy (EIS) and chronopotentiometry at open circuit potential (OCP), the anti-corrosion behavior of the epoxy binder combined with PANI–ZnO nanocomposites was investigated in a 3.5% NaCl solution at a temperature of 25 °C. It was observed that the anti-corrosion capability of pure epoxy coatings was low. The PANI-ZnO nanocomposite-containing hybrid epoxy coating exhibited improved barrier properties and resulted in minimal ion diffusion. Therefore, in corrosive environments, the EPZ2 coating was found to be more protective. Comparing pure epoxy and epoxy/PANI (EP) coatings to the epoxy coating comprising conducting PANI-ZnO nanocomposites (EPZX, where X = 1, 2, 4), it was found that the latter showed lower corrosion resistance and superior barrier qualities in the paint film. The OCP was moved to the noble zone because PANI pigments were included in conducting coatings. On steel substrates, EPZ coatings performed better at preventing corrosion. The resistance value of EPZ2 coatings is roughly three orders of magnitude greater than that of EP coatings and four orders of magnitude higher than that of E coatings. In salty settings, coatings blended with PANI showed undercoating corrosion, whereas coatings blended with ZnO nanorods showed delamination and coating degradation. In another investigation, Navarchian et al., prepared polyaniline (PANI) and polyaniline/clay nanocomposites via in situ oxidative polymerization [42]. Using X-ray diffraction (XRD), the morphology of nanocomposites’ structures was examined. Pigments based on polyaniline were added to epoxy paint, which was then applied to steel substrates. The electrochemical Tafel test, electrochemical impedance spectroscopy, and immersion measurements in NaCl solution were used to examine the impact of the addition of clay and the type of clay cation on the anticorrosion performance of epoxy-based coatings. These cations included Na^+^ in natural clay (MMT) and alkyl ammonium ions in organo-modified montmorillonite (OMMT). The results obtained show that the incorporation of PANI/OMMT nanocomposite into epoxy paint leads to better anti-corrosion properties in comparison with PANI/MMT and neat PANI.

**Figure 4:**
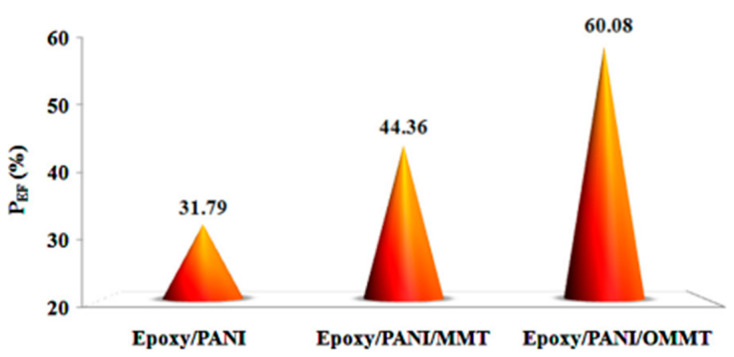
Corrosion protection efficiency values for steel samples coated with epoxy/PANI and epoxy/PANI/clay nanocomposite in NaCl (3.5 wt.%) solution (adopted from reference [42]).

The corrosion protection performance of the epoxy/PANI/clay coatings is significantly higher than that of the epoxy/PANI coating, as shown in Figure 4, demonstrating the silicate layers’ ability to act as a barrier against aggressive species. Conversely, the epoxy/PANI/OMMT coated steel substrate showed a corrosion protection efficiency (P_EF_) value increase of about 16% when compared to the epoxy/PANI/MMT coated surface. Employing electrochemical impedance spectroscopy (EIS) and X-ray photoelectron spectroscopy (XPS) methods, the anticorrosive impact of dodecylbenzene sulfonic acid-doped polyaniline nanoparticles [nPANI (DBSA)] as a conductive polymer was examined in this work [43]. Initially, inverse microemulsion polymerization was used to successfully synthesize n-PANI (DBSA), producing spherical nanoparticles that were found to have an average diameter of less than 30 nm. On the carbon steel substrate, two coating systems were applied: neat epoxy ester (EPE) and a weight percent n-PANI(DBSA) blended epoxy ester (n-PANI(DBSA)/EPE). n-PANI(DBSA)/EPE was prepared by ultrasonicating, using the weight percent of nPANI(DBSA) with EPE. On the carbon steel substrate, n-PANI(DBSA)/EPE and EPE were coated as a single primer. Using EIS measurements in a 3.5% NaCl solution, the anticorrosion performance of the produced coatings was investigated for 77 days. The outcomes unequivocally demonstrated that the n-PANI(DBSA)/EPE coating outperformed the EPE coating in terms of corrosion resistance. This behavior was explained by n-PANI DBSA)’s capacity to release dopant anion when the process of corrosion begins on the metal substrate, highlighting the coating’s clever protection of n-PANI(DBSA)/EPE. Accordingly, a secondary barrier layer that passivates the substrate is created by the released dopant anions and the iron cations. It was studied that based on parametric analysis of the EIS data using the suitable ECMs, due to the inclusion of n-PANI(DBSA) in the coating, all of the protective parameters for the n-PANI(DBSA)/EPE coatings were much superior to those of the EPE coating. In the work of Akbarinezhad et. al, it was observed that supercritical CO_2_ was used to delaminate Cloisite 30B nanoclay in the presence of aniline monomers and by quick mixing polymerization of aniline monomers to prepare exfoliated polyaniline clay (PAniC) nanocomposites with excellent barrier qualities [44]. To enhance the commercial zinc-rich epoxy ’rimer’s (ZRP) initial barrier qualities, the synthesized compounds were applied. After that, the barrier qualities of both modified and unmodified primers were investigated by measuring the electrochemical impedance spectroscopy (EIS) of carbon steel coated panels, the free corrosion potential (Ecorr), and the water vapor transmission (WVT) rate of their free films. The samples treated with PAniC nanocomposites exhibited superior barrier qualities in comparison to the original and modified PAni primers, according to the results. The ZRP formulation successfully modified its barrier characteristics by including PAniC nanocomposites and polyaniline. At the start of immersion, the PAniC modified primer’s coating resistance was at least an order of magnitude higher than that of the other primers. It was also found that the coating resistance of the original, PANI-modified, and PANI C-modified primers was determined to be 267, 1610, and 5540 ohms, respectively, after a year of immersion. Zinc-rich epoxy paint’s protective performance can be altered by adding a tiny amount of PAniC nanocomposites. This will change the paint’s cathodic protection period by enhancing its initial barrier effects and preventing the consumption of zinc powder. To develop conductive scaffolds for nerve tissue engineering, Baniasadi et. al., discussed a method in which highly conductive binary-doped polyaniline nanoparticles and polyaniline/graphene nanocomposites were fabricated using chemical oxidation of aniline via the method of in situ emulsion polymerization in the presence of HCl and sodium dodecyl sulfate (SDS) [45]. Additionally, a modified Hummer’s method was used to create graphene nanosheets, which were then chemically reduced using hydrazine monohydrate. The outcomes, additionally, showed that the electrical conductivity of polyaniline was enhanced from 2 to 7 Scm^−1^ with the addition of less than 1 weight percent of graphene nanosheets to the polymeric matrix. According to the SEM micrographs, the synthesized polyaniline had a spherical shape with a particle size of 10–15 nm, which might be due to the presence of SDS as the emulsifier in the reaction condition. In other words, to create stable, conductive nanocomposites, SDS served as an emulsifier and a dopant agent. The findings of the study on thermal characteristics demonstrated that the thermal stability of the major chains of polyaniline was enhanced by the binary doping method and the addition of graphene to the polymeric matrix. Jafari et al. synthesized PANI/G nanocomposite coatings for corrosion protection of Cu [46]. Cyclic voltammetry was used to successfully electro-deposit PANI/G nanocomposite coatings on Cu in a sulfuric acid solution. It was discovered that PANI/G nanocomposite coatings might indicate a discernible improvement in protection against Cu corrosion process conducted in 5000 ppm NaCl and the degradation rate dropped. The morphology of the PANI/G nanocomposite remained integrated and fault-free after immersion in a 5000 ppm NaCl solution for 120 min, according to a SEM study on PANI/G nanocomposite coated Cu. Graphene nanoparticles were uniformly covered by polyaniline with a likely decrease in the number of polymer pores. It was discovered that the coatings made from PANI/G nanocomposite demonstrated exceptional resistance to corrosion in harsh settings.

#### 4.1.2. Epoxy/Polypyrrole

Due to its electrical conduction properties, environmental stability, and thermal consistency, polypyrrole (PPy) can be used as an intrinsic conducting polymer [47]. Using doping, coatings’ capacity to prevent corrosion has been further improved [48,49,50]. Zinc-rich paints (ZRP) with coatings of PPy-deposited alumina particles (PDAP) were studied by Gergely et al. [51]. They found that the application of highly dispersed PDAPs at a content of 3.2 weight percent and PPy incorporated into zinc-rich hybrids at a content of 0.16 weight percent will lead to the emergence of additional functionalities increasing the long-term anticorrosive performance of ZRPs by scavenging oxidative radicals and inhibiting zinc’s ability to serve as a sacrificial and self-corrosive material, thus preventing the oxidative degradation of the organic binder. The same group created liquid zinc-rich paint and PPy [52]. The hybrid coating with zinc at 80 weight percent and coated alumina inhibitor particles (PCAIPs) at 1.75 weight percent encapsulating PPy at 0.056 weight percent demonstrated the best corrosion protection due to its well-balanced active/passive function. The size-range effect, the spatial dispersal of the alumina-incorporated PPy hindrance particles, and fundamental concepts of the electrical percolation model are used to explain the hybrid paints’ galvanic function. For anti-corrosion protection, PPy has been electrodeposited onto iron and aluminum metal [53,54]. The advantages of epoxy/PPy-based nanocomposite coatings with finely dispersed nanoparticles over the blend include better wear and tensile properties, enhanced corrosion protection, nontoxic behavior, and strong adherence to steel or aluminum alloy substrates. The production of hybrid Ppy–MMT nanocomposites and their impact on enhancing the epoxy coatings’ ability to prevent aluminum corrosion were investigated. It was demonstrated that long-term applications could not benefit from the corrosion protection offered by epoxy blend with MMT (EM) and polypyrrole (EP) systems. The findings demonstrated that the long-term resilience of the epoxy coating is significantly increased when Ppy–MMT nanocomposites are incorporated inside it, as opposed to other coatings. These behaviors are explained by the nanocomposite’s unique shape [55]. By utilizing in situ pyrrole polymerization to insert PPy into MMT layers, MMT/PPy nanocomposites were effectively created. Regarding the ’MT/PPy’s electrical conductivity relative to the PPy.Cl sample, the Cl nanocomposite showed an increase of one order of magnitude, rising from 0.5 S cm^−1^ to 4.4 Scm^−1^. This behavior implies that the MMT serves as a template for the polymerization of PPy, causing the PPy to develop with greater regularity on the forming PPy.Cl chains inserted between MMT layers [56]. PPy/chitosan composites (PCC) with varying feed ratios were successfully synthesized by Kumar et al. using in situ electrochemical polymerization. The PCC coatings were observed to have a consistent and fibre-organized architecture by surface morphological analysis. Adding chitosan moieties to the PPy chain resulted in a significant increase in grain size. PCC, which was made using 50:50 pyrrole (Py): Chitosan (CS), was the most protective PPy/CS coating of those tested [57]. Hosseini et. al. investigated a method using oxalic acid as the supporting electrolyte; in situ electro-polymerization is a simple but effective method for dispersing nanoparticles within a p-type conducting polymer matrix. In harsh settings, coatings composed of polypyrrole–nano-metal oxide particles synthesized by in situ polymerisation were found to have significantly better corrosion resistance than Ppy. The presence of electro-polymerized TiO_2_, Mn_2_O_3_, and ZnO nanoparticles and the anti-corrosion behavior of polypyrrole films in various states on Al electrodes have been studied in corrosive solutions using potentiodynamic polarization and EIS. The effectiveness of polypyrrole films for preventing corrosion on aluminum samples has been shown to significantly enhance with the introduction of TiO_2_ nanoparticles. The polypyrrole formed in the presence of TiO_2_ nanoparticles-wrapped electrodes exhibited a noticeable increase in protection against corrosion processes [58].

#### 4.1.3. Epoxy/Polyurethane

Epoxy/polyurethane coatings for corrosion protection exhibit low porosity, water resistance, and high barrier abilities. As per studies, these coatings are also found to be applied to steel panels [59,60]. Agavriloaie et al. investigated a novel form of polymer concrete made using aggregates and an epoxy polyurethane acrylic resin system. Additionally, it was discovered that adherence stress and pull-out stress were suitable. The coatings’ water adsorption resistance, chemical resistance, thermal conductivity, and thermal shock strength were evaluated [61]. Moradi et al. synthesized a mild steel cathode coated with an aqueous solution composed of a recently developed two-component, one-pack waterborne PU resin using cathodic electrophoretic deposition (CEPD) [62]. Both a blocked isocyanate cross-linker and a hydroxyl-terminated PU prepolymer, which had tertiary amine groups built in to provide the conditions for the synthesis of quaternary ammonium centers under acidic pH, made up the resin mixture. After the electrodeposited components were thermally treated, crosslinked polyurethane coatings with an 80% gel content were formed. An additional improvement to the coating was the addition of a clay component. Promising physico-mechanical qualities were demonstrated by all the produced coatings, including high pendulum hardness, outstanding flexibility, strong impact resistance, and perfect adherence to the mild steel surface. When clean polyurethane coatings were exposed to a 3.5% NaCl solution for seven days, the corrosion inhibition capabilities of the coatings, both with and without co-electrodeposited clay nanoparticles, were evaluated using EIS method. The results showed good corrosion protection. In an article, Sharmin et al. studied epoxidized linseed and *Pongamia glabra* oils, as well as the products developed from them, such as epoxy-polyols, epoxy-polyurethanes, and epoxy-polyurethane coatings. The coatings intended to prevent corrosion illustrated significant antibacterial efficacy against Escherichia coli [63]. Using sodium p-sulfonatocalix [4] arene (SC4A) and p-tert-butyl calix [4] arene (BC4A) modified graphene oxide nanosheets (SGO and CGO) as green corrosion protective coating, Mohammadi et al. investigated the preparation of novel environmentally friendly aqueous dispersions of polyurethane nanocomposites [64]. The results of the anti-corrosion evaluation showed that waterborne polyurethanes (WPU)/CGO have the best anti-corrosion activity due to their increased barrier property against electrolyte diffusion and hydrophobicity, which inhibits water absorption. With a notable 99.8% inhibition efficacy, this new aqueous polyurethane nanocomposite dispersion can be regarded as an effective anti-corrosion coating for mild steel. Dodecyl benzene sulfonic acid doped with polyaniline was included in epoxy and polyurethane coatings developed by Diniz et al. [65]. The applied coatings on steel plates showed a rise in capacitance and a decrease in electrical resistance, determined by electrochemical impedance spectroscopy.

#### 4.1.4. Epoxy/Poly(vinyl alcohol)

PVA, or poly(vinyl alcohol), is a benign polymer that can generate films. PVA encourages the manufacture of anticorrosion products with a cleaner approach. This polymer may also attach to the surface of the metal [66,67]. Graphene-blended PVA nanocomposites for effective corrosion protection for an aluminum 2219 alloy. Using an electrochemical workstation, the corrosion-preventing properties of pure PVA and graphene-impregnated PVA-based surface layers on aluminum were examined. The GPVA-coated aluminum exhibits better corrosion resistance properties than the bare aluminum, as confirmed by the EIS [68]. The ability of PVA coatings to avert corrosion on mild steel testing panels has been investigated in a variety of aggressive situations at various loading levels of GO nanoparticles. In comparison to uncoated mild steel panels, the electrochemical and non-electrochemical corrosion studies of the coated panels demonstrated that PVA polymer matrix filled with 3.0 weight percent GOPVA provides superior protection in 0.5 M HCl and 3.5% NaCl [69]. To improve graphene’s compatibility with epoxy resin, Ding et al. reported the synthesis of a new hydroxyl PGHEP as an effective dispersion [70]. They observed that based on the results of salt spray tests and EIS, the PGHEP functionalized graphene sheets dispersed composite coatings showed improved corrosion resistance when compared to pure epoxy resin. These coatings also had a higher contact angle and lower water absorption. The generated passive film from evenly dispersed PGHEP functionalized G sheets, which function as a physical barrier on the steel surface, was primarily responsible for the better corrosion protection performances of G/epoxy coatings. Ramezanzadeh et al. reported that zinc phosphate conversion coating at room temperature was applied to steel substrates upon chemical treatment. To prevent green corrosion, PVA was added to the phosphate solution [71]. Lastly, the surface-treated and untreated steel samples were coated with epoxy/polyamide. The findings indicated that PVA added to the phosphate coating resulted in a decrease in phosphate grain size and an increase in phosphate crystal population density. Compared to a Zn-treated steel surface, the Zn-PVA treated steel surface had a smaller contact angle. On the steel substrate treated with zinc phosphate conversion coating including PVA, the corrosion resistance of the epoxy coating was significantly enhanced. Additionally, PVA made the epoxy coating more adherent to the steel surface and considerably reduced cathodic delamination. On the one hand, the polar water transport route allows the corrosive medium to diffuse to the metal-coating interface, where it can corrode the metal. Conversely, a lot of hydroxyl groups can establish hydrogen bonds with water molecules, which can reduce molecular proximity and cause polymer expansion. The same metal corrosion protection effect as solvent type anticorrosive coatings is currently impossible to obtain with PVA waterborne anticorrosive coatings [72]. The same metal corrosion protection effect as solvent-type anticorrosive coatings is now impossible to obtain with PVA’s aqueous anticorrosive coating. Because of their strong interface compatibility, PANI, graphene and its derivatives, and nanocellulose were doped into the PVA coating matrix to increase the corrosion resistance of PVA [68,69,73,74,75]. The resulting nanocomposite coatings demonstrated good mechanical and anticorrosion capabilities [76].

#### 4.1.5. Epoxy/Polyester

Pure epoxy resins and a blend of epoxy and polyester resins can be used for the manufacture of anti-corrosion coatings with nanocomposites [77]. Mirabedini et. al., developed a method to increase the effectiveness of epoxy/polyester powder coatings for corrosion protection. The high ohmic resistance of the coating both before and after saturation with water is responsible for the powder coating’s comparatively good performance, as seen by the impedance data. Additionally, the data showed that the degreased samples functioned better compared to the polyacrylic acid (PAA)-treated samples. After 60 days of immersion, samples treated with PAA + H_2_ZrF_6_ (PZr) demonstrated corrosion inhibition. Even after the test was finished, chromate/phosphate conversion coated (CPCC) samples showed evidence of corrosion protection [78]. In recent years, photo-curable resins that are based on multifunctional acrylate monomers have also been utilized to fabricate bulk items like dental fillings and 3D-printed parts. These resins are often applied as thin films, such as protective coatings and printing inks. These systems include brittleness and low impact resistance caused due to the inhomogeneous polymer architecture and high crosslink density, but they also have advantages like quick curing and strong spatial resolution [79]. Morteza et al. investigated to develop an epoxy-based nanocomposite to improve corrosion protection properties by using various combinations of graphene oxide (GO) and Cloisite 20A montmorillonite (MMT) nanoclay (NC) particles functionalized with aminosilane and 1,4-butanediol diglycidyl ether (BDDE) molecules [80]. On the corrosion resistance of epoxy coating, the impacts of modified NC (MNC), modified GO (MGO), and various MNC-MGO mixtures were investigated. It was observed that the stability of surface-modified GO and NC particles in the epoxy matrix was improved. Comparing the epoxy coating with clean particles, the corrosion resistance was significantly improved by incorporating MNC and MGO particles. The coatings containing MGO particles demonstrated a greater level of corrosion resistance in comparison to those containing MNC particles. The combination of MNC-MGO at a ratio of 30:70 *w*/*w* was shown to increase the coating barrier and corrosion protection qualities the most. Sari et al. found in a study that the surface of the nanoclay particles was modified using different concentrations of hyperbranched polyester-amide polymer (HBP) [81]. The effectiveness of the HPB grafting on the clay particles was evaluated using thermal gravimetric and X-ray diffraction analyses. To create epoxy/clay nanocomposites, one weight percent of both modified and unmodified clays was added. The nanocomposites’ ability to prevent corrosion was assessed through the use of electrochemical impedance spectroscopy (EIS). The results showed that when the “polymer/clay” ratios were 10/1 and 5/1, in particular, the surface modification of the clay particles by HBP significantly increased the corrosion resistance of epoxy coatings.

#### 4.1.6. Epoxy/Polyamide

Because epoxy coatings are so durable, adhere well to metal substrates, and are exceptionally tough, they are frequently employed to shield metal structures from corrosion and environmental threats. Consequently, epoxy coatings containing fillers have been created to improve metals’ resistance to corrosion [19,32]. Golru et al., developed epoxy/polyamide nanocomposites coatings at different loadings of nano-alumina particles [20] on the AA1050 metal substrate to study the effects of nanoparticle addition on the nanocomposites’ resistance to corrosion and hydrolytic degradation. The findings demonstrated that addition of nanoparticles enhanced corrosion resistance and even at large loadings, the nanoparticles are distributed evenly throughout the coating matrix. The enhanced corrosion resistance was evident from the salt spray and EIS test results. Comparing the epoxy nanocomposites to the blank sample, nanocomposite coatings exhibited higher capacitive behavior. Nikravesh et al., showed the corrosion resistance of an epoxy-polyamide coating enriched with various volume ratios of micaceous iron oxide (MIO)/Al pigments [82]. Using MIO and Al pigments, the coating’s resistance to corrosion was enhanced. The corrosion resistance of the coating reinforced with 100% Al particles was found to be substantially greater than that of the coating reinforced with 100% MIO particles. The sample supplemented with 10% MIO and 90% Al particles showed the highest improvement in the coating’s anti corrosion property. Using MIO particles at greater concentrations resulted in an inappropriate corrosion resistance of the coating. Al particles were much more capable of interacting with the OH ions than MIO pigment was. This may be the cause of the precipitation of aluminum hydroxide on the steel surface, which increases the coating’s resistance to corrosion. Ramezanzadeh et al. observed that at low concentrations of ZnO nanoparticles, a uniform distribution of nanoparticles (at low loadings) was established at the surface and bulk of the epoxy-polyamide coating [83]. At large loadings, it was found that nanoparticles tend to form some agglomerations and migrate to the majority of the coating. At low nanoparticle loading, an increase in T_g_ and a decrease in cross-linking density are observed. The epoxy coating’s curing behavior was adversely affected by nanoparticles. Changes in the coating’s degree of curing brought about by nanoparticles result in a decrease in Tg and cross-linking density. ZnO nanoparticles greatly increased the epoxy coating’s resistance to corrosion. To improve the protective and self-healing properties of epoxy/polyamide coatings for mild carbon steel, Zhang et al. investigated that the addition of epoxy microcapsules and polyaniline nanofibers improved the protective and self-healing properties of the same [84]. The widely recognized method of interfacial polymerization is used to create epoxy microcapsules. The model corrosion media is a solution of sodium chloride (NaCl, 12 weight %). Due to the encapsulate epoxy’s controlled release and the passive nature of PANI nanofibers, these materials exhibit exceptional protective performance and self-healing behavior. The outcomes may shed some light on how to prepare protective coatings with excellent performance. Mirzakhanzadeh et al., in their study, observed that 2-mercaptobenzimidazole (MBI) and zinc aluminum polyphosphate (ZAPP) worked together to protect mild steel covered in a solvent-borne epoxy-polyamide layer from corrosion [85]. The combined inhibitors exhibit better corrosion protection than those containing either ZAPP or MBI, as evidenced by the trend and magnitude of electrochemical impedance spectroscopy data collected after a 70-day immersion in a 3.5-weight % NaCl solution. Pull-off experiments demonstrate that the enhanced adhesion strength is provided by the combined inhibitor system. According to SEM and electrochemical research, the formation of a protective layer at the coating/metal interface after exposure to electrolytes is associated with improved corrosion performance.

#### 4.1.7. Epoxy/Poly(dimethylsiloxane)

Poly(dimethylsiloxane) (PDMS) and epoxy are two distinct polymers that are employed as multifunctional anticorrosion coating systems [86,87]. In organic coatings, silicon nitride was initially employed as an anti-corrosive pigment. Furthermore, an efficient method was suggested to improve the dispersibility of silicon nitride in epoxy resin and apply it to protect Q235 carbon steel by mixing inorganic fillers and organosilanes. Two factors contributed to the improved silicon nitride coating’s strong anticorrosion performance, according to the results. KH-570 was utilized, on the one hand, to enhance the potential chemical interactions between epoxy resin and silicon nitride particles [88]. Zhang et al. studied that the soft lithography based on a PDMS template was used to create a superhydrophobic epoxy covering with highly reproduced surface microstructures from lotus leaves. Wet–dry cyclic immersion, fluid immersion, and salt spray tests were the three corrosion tests in which electrochemical measurements were made. The results were compared to those obtained for standard smooth epoxy coating. The superhydrophobic coating’s extremely capacitive behavior and significantly improved barrier performance compared to the conventional coating was attributed to the air films trapped in its surface microstructures under immersion and cyclic immersion circumstances. The salt spray test produces ultrafine electrolyte particles, which are easily able to pass through the microstructure and make direct contact with the coated surface. As a result, the air films did not form, and the superhydrophobic coating began to deteriorate more quickly [89].

#### 4.1.8. Epoxy/Polythiophene

The physical properties of polythiophene (PTh) and its derivatives are excellent [90,91]. In addition, polythiophene has good conductivity, environmental resilience, and specific surface area. Many applications, including sensors, supercapacitors, batteries, and electrical devices, have used polythiophene [92].

Polymers, when combined form polymer alloys, result in interpenetrating polymer networks (IPNs) that are found to exhibit controlled morphologies and synergistic behavior. Palraj et al. showed the preparation of corrosion-resistant IPNs from immiscible resins (epoxy, silicone, and thiophene) by using a cross-linking agent and a catalyst. Studies such as GPC, FTIR, NMR, TG, DTA, and SEM were used to fix the best performing IPN. It was confirmed using surface morphology studies and SEM that the incorporating silicone and polythiophene into the epoxy polymer resulted in the formation of homogeneously micro structured IPN. TG-DTA studies revealed that there is enhancement in the heat resistance properties of the modified IPN. The heat resistance property of the IPN displayed an upper stability limit of 380 °C for IPN with optimum silicone content and, with higher silicone content, it reached up to 325 °C for IPN [25].

### 4.2. Nanocomposite Polymer Coatings for Anti-Corrosion Other Than Epoxy-Based

#### 4.2.1. Polyaniline (PANi)-Based Nanocomposite

**Figure 5:**
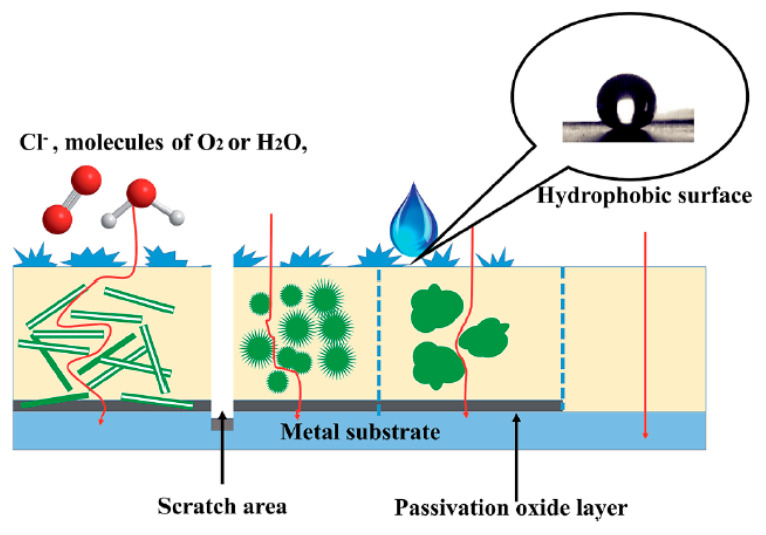
Schematic depiction of the mechanism of PANI nanostructure-based coatings (adopted from reference [98].

PANis are the most investigated polymers owing to their exclusive electronic, optical, photovoltaic, thermal, sensing, electrical, mechanical, and structural properties, environmental stability, unique redox behavior, and, most importantly, their facile synthesis [93]. The much-dominant polyaniline-based coatings are commonly accepted to prevent corrosion by suppressing the permeation of corrosive agents and concomitantly allowing the formation of passive oxide layer on the metal surface [94]. PANi can be used as a protective coating directly electrodepositing on the metal surface, by electrochemical polymerization of aniline monomers [95,96]. However, PANi, on electrodeposition, revealed some limited practical applications because of its porous structure and poor adhesion to metal substrate [97]. In order to make it a potent corrosion protector, PANi was generally blended in polymeric resin as an additive to form composite coatings, which blends the good adhesion and barrier properties of organic coatings and the electroactive properties of PANi [98]. Figure 5 shows that the nanostructure of PANI composites enhances coating adherence and mechanical properties by improving interactions between the nanoscale composites and the target substrates. PANI nanostructures extend the paths for Cl, H_2_O, and O_2_ to reach the metal substrate, improving the coating’s ion and gas barrier characteristics. Polyaniline (PANi), the widely employed additive used for organic coatings affording corrosion resistance, can be embedded in epoxy, as a pigment in a clay composite, and also act as an anti-corrosive agent to protect steel [99].

Qiu et al. fabricated self-doped sulfonated polyaniline (SPANi) nanofiber by carrying out the copolymerization of ASA and aniline through an approach of rapid mixing polymerization. As investigated by the scanning electron microscope (SEM) and transmission electron microscope (TEM), the prepared SPANi nanofibers revealed an average diameter of 45 nm and length of 750 nm. The corrosion protection properties of waterborne epoxy coating as proven by the polarization curves and electrochemical impedance spectroscopy (EIS) rose to a very high standard by the incorporation of only 0.5 wt.% SPANi nanofiber. This can be attributed to the ultra-small nanofiber structure and unique reversible redox behavior of SPANi. As per the XRD and SEM studies, it was revealed that the inclusion of SPANi nanofiber in the epoxy coatings expedited the formation of a passive layer, consisting mainly of Fe_2_O_3_ and Fe_3_O_4_ on the steel surface [100].

Superhydrophobic polyaniline (PANI)/polystyrene (PS) micro/nanostructures were synthesized by drop casting the PANI micro/nanostructure on electrospinning PS nanofiber mats and using it as an anticorrosion coating for protecting carbon steel. Investigation and comparison on the fabricated PANI/PS micro/nanostructures in 0.1 M H_2_SO_4_ using electrochemical impedance spectroscopy (EIS) and potentiodynamic polarization technique displayed its excellent corrosion resistance ability and durance property. Y. Zhao et al. found that PANI/PS micro/nanostructures can provide promising anticorrosion performance for carbon steel, and there is an increase in the efficiency of corrosion protection (η) along with the water repellency of the PANI/PS micro/nanostructures. As the PANI-PFOA micro/nanostructure sticks properly on PS microfibers, the coating cannot easily shed off. The synthesized PANI-PFOA/PS micro/nanostructures show a water contact angle value as high as 153°, which exemplifies the magnificent anticorrosion properties with a promising anticorrosion efficiency of 99.48%. Thus, mingling on the multiple effects proves to be advantageous in efficaciously protecting the metallic substrates against corrosion [101].

Owing to the electrochemical and conducting properties of intrinsically conducting polymers (ICPs), they have been used in the coatings industry [102,103]. Though the ICPs are equipped with good chemical and electrical properties, they tend to have poor mechanical strengths, which can be made better through the incorporation of inorganic clays such as montmorillonite (MMT) in the polymer [104]. MMT is a phyllosilicate clay mineral which is characterized by stacked layers of aluminum octahedrons and silicon tetrahedrons. In situ synthesis of PANI/clay composite was executed through the oxidative polymerization technique. PANI/clay composite particles dissolved in NMP by ultra-sonication followed by mixing into an epoxy resin formulated the coating. Further, the addition of clay improved the BET (Brunauer–Emmett–Teller) surface area of PANI. The corrosion protection rate improved as observed through immersion testing and electrochemical studies. MMT, when well-dispersed in the composite, induced superior properties because of slower diffusion compared to that of the pristine PANI and ex situ PANI/clay matrixes. The anticorrosion properties are significantly influenced by the level of crosslinking. The steel substrate is protected by epoxy coatings; however, when modified with PANI, there has been a significant improvement in the corrosion protection properties. PANI/clay evidences a higher corrosion rate compared to that of pristine PANI, which can be attributed to agglomeration, as proven by the SEM images of the surface morphologies of the coated metals. It is the crosslinking and poor wet ability of the PANI/clay composite that lessens the rate of ion exchange, thereby improving the corrosion protection of the coated metal. Thus, modulating epoxy with a conducting PANI composite improves both its mechanical and corrosion-protection properties compared to the neat epoxy [99]

The group reports a novel polyimide (PI) and polybenzimidazole (PBI)-based system synthesized and reinforced with two types of nanoparticles, i.e., polyaniline and carbon nano-onion. In situ generation of Polyaniline nanoparticle was performed in polybenzimidazole (PBI-in-PANI). The remarkable nanofiller used here was carbon nano-onion (CNO) which significantly affected the morphological, conducting, and corrosion properties of novel coatings. A fine network-like morphology for PI/PBI-in-PANI/CNO nanocomposite was revealed by FESEM studies. As diagnosed by TEM studies, in the carbon nano-onion reinforced polyimide matrix, homogeneously dispersed spherical nanoparticles with well-defined boundaries have been observed, that is, small spherical PBI-in-PANI nanoparticles and CNO in the matrix were revealed. Studies showed that the inclusion of CNO and enhancement in temperature increased the electrical conductivity properties concomitantly, decreasing the percolation threshold of the nanocomposites up to 5% loading. A significant increase in the corrosion protection effect up to 70% was seen when the composition of the nanocomposite was 5% PI/PBI-in-PANI/CNO [105].

Khan et al. demonstrated the use of waste ferrochrome slag (FeCr-slag) for corrosion protection. The nanostructured material was obtained by the milling of FeCr-slag for a tenure of four hours. The prepared nanomaterial was also used to make a nanocomposite with polyaniline by the in situ polymerization technique. Morphology examinations of FeCr-slag revealed the homogeneous dispersion with minor agglomeration and the diameter of about 80–100 nm with spherical morphology. In contrast, the microstructural observation of nanocomposite implied that the FeCr-slag nanoparticles are well embedded and strongly held to the PANI matrix, displaying the presence of some interaction between the FeCr-slag nanoparticles and the PANI matrix. The evaluation of the performance of the anticorrosion behavior of FeCr-slag material, PANI, and a nanocomposite of FeCr-slag material with polyaniline was carried out by invoking these pigments in a commercial epoxy zinc rich phosphate primer. The investigation of the anticorrosion ability for the prepared coatings was executed by employing the electrochemical potentiodynamic polarization (PDP) and electrochemical impedance spectroscopy (EIS) methods and their exposure to corrosive media in salt spray chamber. As per the results obtained, the nanocomposite pigments performed better, followed by the FeCr-slag nanomaterial, followed by PANI, respectively. This is attributed to the fact that the incorporation of PANI into the coatings performs as a barrier that inhibits the permeation of the electrolyte, thereby lessening the corrosion rate by participating in the electrochemical reactions. Thus, the addition of the conductive polymer PANI has significantly improved the anticorrosion properties of the paint system used [106].

A Nb:TiO_2_ nanofibers/PANI composite coating was fabricated through the galvanostatic method on 316 stainless steel for bipolar plates of a proton-exchange membrane fuel cell. The corrosion potency of the composite coating was investigated in the simulated cathodic and anodic environments (0.3 M HCl bubbled with air or H_2_ at 25 °C) by the potentiostatic polarization, potentiodynamic polarization, open circuit potential, and electrochemical impedance measurements. The experimental results indicated that the corrosion current density of the composite coating coated 316 stainless steel in both the simulated cathodic and anodic environments decreased by an order of magnitude to about 2.75 and 6.31 μA·cm^−2^, respectively; the corrosion potential increased by more than 400 mV vs. SCE. During the 180 h immersion, the composite coating, with a compact structure and few micropores, reduce the adsorption and infiltration of the corrosive ions into the coating, displayed escalated chemical stability, and showed effective protection for the 316 stainless steel with a better physical barrier effect and in situ anodic protection effect [107].

Lei et. al., in the year 2019, fabricated PANI/CeO_2_ nanocomposites (NPs) by in situ polymerization of aniline in the presence of CeO_2_ NPs. The synthesized coatings from the fabricated PANI/CeO_2_ NPs displayed excellent corrosion resistance, which proved to be superior to that of epoxy coatings added with PANI in NaCl solution. Variegated analytical facilities, i.e., powder XRD, FT-IR, SEM, and high-resolution transmission electron microscopy (HRTEM), were employed in order to characterize the synthesized CeO_2_ and PANI/CeO_2_ NPs. Based on the test results of EIS, salt spray, and LEIS, though the epoxy coating was found to have the ability to provide corrosion protection to the substrate due to its good barrier effect, the coating showed an inability to stop or delay corrosion when localized defects were present. Thus, this coating, upon prolonged exposure, becomes damaged. The PANI/CeO_2_/epoxy coating shows higher stability and protective performance in spite of the presence of the defects in the coating, suggesting that the corrosion activity is pre-emptively healed and cannot proceed. The noteworthy improvement of the corrosion protection performance of the PANI/CeO_2/_epoxy coatings is related to the synergetic protection of the increase in the protective barrier due to the role of CeO_2_ NPs and PANI against the diffusion of aggressive ions (e.g., Cl^−^) and the improvement of self-healing protection attributed to the redox behavior of PANI. Therefore, the hybrid PANI/CeO_2_ NPs are considered as one of the best pathways in order to escalate the protection performance of the epoxy coatings on carbon steel [108].

An approach towards the synthesis and characterization of polyaniline (PANI) and two modified loadings of 5 and 10% Tl_2_O_3_-SiO_2_/PANI involves Tl_2_O_3_-SiO_2_ nanoparticles (NPs) modified with PANI forming nanocomposites. Studies like X-ray diffraction (XRD), UV–vis absorption spectra (UV–Vis), Fourier transform infrared spectroscopy (FTIR), scanning electron microscopy (SEM) with energy dispersive X-ray spectroscopy (EDX), high-resolution transmission electron microscopy (HR-TEM), and the dynamic light scattering (DLS) technique confirmed the formation of the composite and its structure. The HRTEM results implicated that the hexagonal mesoporous arrangement of SBA-15 was not affected by the incorporation of PANI, which has been confirmed by the HRTEM results.

The inhibition efficiency obtained by the investigated PANI and Tl_2_O_3_-SiO_2_/PANI nanocomposites for the corrosion of C-steel in the studied aggressive solution at 150 ppm is as follows: 10% Tl_2_O_3_-SiO_2_/PANI > 5%Tl_2_O_3_-SiO_2_/PANI > PANI. Zeta potential analysis emphasizes the higher stability of T-S/PANI nanocomposite suspension. EIS and PDP measurements concluded the synthesis of a protective film of PANI and T-S/PANI nano composite on the C-steel/HCl solution interface. With an increase in the NCs dose, the percentage of efficiency of the nanocomposite also increases, but it decreases with increasing the temperature. The protection potency of PANI film on C-steel was increased by the insertion of Tl_2_O_3_-SiO_2_ in the nanocomposite. The Tl_2_O_3_-SiO_2_ nano particles also played the role of a physical barrier against attacking of corrosive ions. The adsorbed film over the C-steel surface was also substantiated by SEM/EDX analysis. The deposition of PANI into the Tl_2_O_3_-SBA-15 channels provides a good protective layer on the steel surface [109].

Button-shaped polyaniline (PANI) and two-dimensional graphene oxide (GO) nanosheets were used to fabricate an environmentally friendly, nontoxic composite that was then modified by polydopamine (PDA).

In this study, the composite of two-dimensional graphene oxide (GO) nanosheets and button-shaped polyaniline (PANI) was synthesized and further modified by polydopamine (PDA). The obtained PDA-PANI-GO composite was used to enhance the corrosion protection ability of nontoxic water-based alkyd varnish (WAV). The chemical composition, functional groups, and surface morphologies of GO, PANI-GO, and PDA-PANI-GO composites were characterized by XRD, FT-IR, XPS, and SEM. The anticorrosion performance was demonstrated by electrochemical impedance spectroscopy measurements and polarization tests. Due to the physical barrier effects and surface hydrophobicity of PANI-GO composite, the approaches of the caustic substances to the surface of the metal were inhibited, while the highly adhesive PDA molecules reinforced compatibility between fillers and WAV. The results showed that PDA-PANI-GO composite introduced WAV-enhanced corrosion prevention performance. Under the optimal conditions, where the ratio of PDA to PANI-GO was kept at 2:1, the impedance values escalated by over two orders of magnitude compared with bare steel. The study can pave out a pathway to reduce the corrosion of metals especially for situations such as buildings and equipment in coastal areas and the outcome of this study would assist in decreasing the costs for engineering construction and maintenance, and, at the same time, reducing the release of toxic heavy metals [110].

In order to serve as the protective barrier for metallic substrates against corrosive environment, commercial paints and coatings are used. For achieving both barrier and active protection, a considerable variety of nanostructures can be embedded. Haddadi et al. aimed to demonstrate the role of polyaniline (PANI) as an active polyelectrolyte modifier used for the remodeling of mesoporous silica nanoparticles (MSNs) doped with zinc cations (Zn^2+^). Investigation of the influence of MSNs before and after coverage with Zn^2+^ doped PANI on the rheological, mechanical, and corrosion protection properties of the epoxy coating was carried out. Different techniques such as field emission scanning electron microscopy (FE-SEM), transmission electron microscopy (TEM), N_2_ adsorption-desorption, Fourier transform infrared spectroscopy (FTIR), Raman spectroscopy, X-ray diffraction (XRD), thermogravimetric analysis (TGA), inductively coupled plasma optical emission spectroscopy (ICP-OES), rheometric mechanical spectroscopy (RMS), differential scanning calorimetry (DSC), tensile testing, and electrochemical impedance spectroscopy (EIS) were employed for characterizing the fabricated samples. The formation of PANI shells onto the surface of silica cores and pH triggering the release of Zn^2+^ at the alkaline condition was proved through the characterization of PANI-MSNs. Rheological and mechanical analyses displayed that the coverage of MSNs with PANI improved the rheological properties and mechanical characteristics, i.e., the yield stress and Young’s modulus of the epoxy coatings. The electrochemical measurements demonstrated that the incorporation of 3 wt.% PANI-MSNs in the epoxy coating escalated the active corrosion protection and barrier properties of the resulting nanocomposite coating. As per the studies carried out, the modification of MSNs with PANI and doping of the nanoparticles with Zn^2+^ as an eco-friendly inorganic corrosion inhibitor served the purpose for the enhanced dual active/barrier characteristics [111].

#### 4.2.2. Graphene Oxide (GO)-Based Coating

GO consists of hexagonally arranged sp^2^ and sp^3^ hybridized carbons. The surface of graphene consists of the hydroxyl, epoxide, carbonyl, and carboxyl functional groups. GO provides better matrix/nanofiller interactions and hydrophilicity because of the presence of the oxygen functional groups. Owing to the unique lamellar structure of graphene, it is potent to prevent the diffusion of oxygen and water molecules to the surface of meta-based materials, thereby protecting the metal from oxidation corrosion [112]. Graphene and its derivatives have been propitiously used as preservative additives to boost the corrosion resistance of coatings [113,114].

With a view to designing new surfaces possessing the dual properties of hydrophobicity and corrosion resistance, multilayer sol–gel nanocoating of graphene oxide onto aluminum alloys has been fabricated by Maeztu, J.D et al. The choice of a multilayer hybrid sol–gel matrix is to have a good entrapment of the GrO_x_. In order to increase the hydrophobicity, a new multilayer simple sol–gel coating has been incorporated into the previous hybrid matrix by the incorporation of fluorinated polymeric chains in the outer surface of the nanocoating. The thermal treatment causes the formation of harder films with a good adhesion due to the formation of a highly crosslinked sol–gel network, resulting in enhancement of the resultant hydrophobicity [115].

Hayatgheib et al. functionalized GO with PANI nanofibers through three variant methods. Out of the three methods used, in the second method, aniline polymerization was carried out using SDS as a surfactant and ammonium persulfate as an initiator. However, in method I and in method III, the surfactant and the initiator were removed during the polymerization procedure respectively. Epoxy nanocomposites were fabricated through inclusion of pure GO and the three varying types of GO/PANI nanosheets (obtained from methods I to III) via a wet transfer method (WTM). EIS studies the corrosion protection performance of the nanocomposites on steel substrate, which revealed that GO PANI notably improved the barrier performance and provided active inhibition for epoxy coating, that is, the deposition of PANI nanofibers on the GO sheets significantly enhances their compatibility with epoxy matrix. The elevated amounts of PANI nanofibers deposited on the GO surface obtained from the nanosheets’ structure and composition were described. After that, the GO/epoxy and GO-PANIs/epoxy nanocomposites were prepared, and EIS examined how well they protected against corrosion on a steel substrate. GO PANI significantly enhanced the barrier performance and offered active inhibition and ionic resistance for epoxy coatings, according to the results. The elevated amounts of PANI nanofibers deposited on the GO surface obtained from method I resulted in the best interaction and dispersion properties. PANI layers on the GO surface enriched the storage modules and cross-linking density and enhanced the T_g_ of the epoxy coating by participating in curing reaction with epoxy reactive sites. The ionic resistance of the epoxy coating enhanced with the incorporation of PANI nanofibers by providing a positive surface charge on the GO sheets and decreasing the migration of the hydrated Na^+^ cations to the cathodic regions. Thus, the PANI film increased the dispersion of the GO sheets and, hence, escalated the diffusion length for the corrosive electrolyte [116].

Polymeric compounds have been investigated as powerful and affordable corrosion inhibitors that form a protective layer on the metal surface and stop it from dissolving in corrosive solutions. A corrosion inhibitor is a material that, when added to a corrosive environment in small amounts, reduces the rate of corrosion [117]. The anti-corrosive potency of NSP-GO on two synthesized water-soluble polymeric compounds, namely, melamine formaldehyde (MF) and urea formaldehyde (UF), were examined in the saline environment. The inhibition efficiency of urea formaldehyde (UF) and melamine formaldehyde (MF) was raised from 93% and 95%, respectively, to ~100% for both composites as per the potentiodynamic polarization and EIS results, with the incorporation of NSP-GO at 500 ppm concentration. The combined effect of graphene oxide nanosheets’ great surface-covering ability and heteroatoms’ strong attraction for metallic surfaces is responsible for this increased level of protection. The MF and UF polymer films serve as complete defenders because the heteroatoms in the decorated GO sheets act as anchors, drawing the GO sheets towards the metal surface. The SEM data unequivocally demonstrated that employing the composite polymeric compounds reduced the pit number and the extent of the damage [118].

GO was potentially used as a surface-active agent to improve nanoclay (NC) exfoliation in a phenol novolac glycidyl ether to achieve superior anti-corrosion performance. In order to establish this, different levels of GO/NC were built and impregnated into a phenol novolac epoxy resin. The FT-IR, Raman spectroscopy, and FE-SEM methods were used for particle characterizations. Also, the conclusions from the various experiments showed the escalated potency of the GO sheets to be adsorbed on the clay lamellae surface, behaving as a surfactant and giving rise to an efficient clay particle dispersion in the phenol epoxy film. The results of the corrosion study of the un-scratched coatings showed that using the proper mixture of clay particles and GO sheets (i.e., G/N:20/80/EPh and G/N:34/66/EPh samples) resulted in significant improvement in the epoxy coating corrosion protection function even after prolonged immersion time. As per the salt spray test results, the G/N:43/57/EPh outperformed G/N:50/50/Eph, but from the EIS results of the scratched samples, the G/N:50/50/EPh showed better protection than G/N:43/57/EPh. Pull-off adhesion and cathodic disbondment test (CD) test results displayed that the clay particles dispersion improvement in the presence of GO sheets caused in the lower coating delamination after the cathodic disbondment test and higher adhesion strength values. The better coating barrier performance because of the synergic effect of G/N (especially at 20/80 and 34/66 ratios) was credited to the better dispersion of the particles [119].

The recent scenario has an exploding interest towards the incorporation of graphene-based nanomaterials for protection of corrosion. Keeping this in view, tannic acid, as a green reducing agent, was employed for TA-rGO to offer promising nanoplatforms with decent dispersion in an epoxy matrix for heightened mechanical and anti-corrosion properties. Further, enhancement of the active corrosion protection by ~71.5% was noted through EIS studies, when these nanoplatforms were doped with cerium cations (Ce@TA-rGO). It also even presented excellent barrier properties without electrolyte diffusion after 10 weeks of immersion. Higher cross-linking densities and tensile strengths were demonstrated by Ce@TA-rGO-EP and TA-rGO-EP nanocomposites in comparison to unfilled epoxy in terms of mechanical properties. This was due to an improvement in the degree of dispersion and interaction of nanoplatforms with the polymer [120].

Bahlakeh et al. studied the impact of the surface treatment of steel substrate when treated with a nanostructure cerium–lanthanum conversion coating (Ce–La CC composite film) by imparting its effect on the interfacial adhesion and corrosion resistance of a melamine-cured polyester coating (MCPC). Surface roughness and surface free energy are found to be improved when a homogeneous, crack-free Ce–La composite layer is deposited on the steel surface. The interfacial adhesion strength of MCPC was significantly increased when Ce–La CC was present. When compared to the untreated sample, the one treated with Ce–La composite film exhibited lower adhesion loss. Melamine-crosslinked polyester resin adheres to untreated or treated steel layers via electrostatic and Lewis acid–base interactions, according to computational studies based on MD and MC simulations and QM calculations. Additionally, the coating has a stronger adhesion to Ce-/La-covered steel sheets. Even in damp situations, the conversion layer’s superior protective efficacy was demonstrated by the investigation of coating adhesion strength in wet conditions [121].

Layered double hydroxides (LDH) materials, similar to graphene, have a unique lamellar structure having high metal dispersion, a high surface area, and thermal stability [122].

In the current scenario, nanocomposites based on graphene and LDH materials are commonly used as supercapacitors and catalysts [123]. Also, based on the earlier studies, it was confirmed that there is an established synergistic effect between rGO and the LDH layer [124].

Following an effective one-step synthesis of the reduced graphene oxide zinc aluminum layered double hydroxides (rGO-ZnAl-LDH) micro nano fillers, 3-aminopropyl triethoxysilane was used for modification. This was then added to a water-borne epoxy (EP) matrix to create the ZnAl-LDH/EP composite coating. The goal of adding 3-aminopropyl triethoxysilane was to increase the corrosion resistance of EP coating by functioning as a functional filler. Investigation through the potentiodynamic studies, electrochemical impedance spectroscopy (EIS), and salt spray tests have shown that the ratio of GO:ZnAl-LDH and the adding amount of M rGO-ZnAl-LDH in EP has an influence on the anti-corrosion resistance. SEM images demonstrated that the sample with a ratio of rGO:ZnAl-LDH (2:1) has the least porous surface, which remarkably improved the anti-corrosion resistance. As suggested by the electrochemical studies, the best anti-corrosion resistance with the lowest corrosion current density (0.0733 μA/cm^2^) and the largest coating resistance (2.77 × 10^4^ Ω cm^2^) was found when the adding amount of rGO:ZnAl-LDH (2:1) into EP was 0.5 wt.% [125].

#### 4.2.3. Zeolitic Imidazole Framework Nanoparticles

Amongst the emergent class of functional materials with promising nanostructures and applications in biomedicine and materials science, the zeolitic imidazole framework (ZIF) holds a unique position. Owing to the high content of corrosion inhibitor in the ZIF-7 nanoparticles and its pH-sensitive nature, the ZIF-7 nanoparticles quickly respond to the surrounding acid environment and emancipate the linker as healing agent, leading to a new barrier on scratched metal surfaces. Yang et al. demonstrated the four types of ZIF-7 nanoparticles, i.e., ZIF-7-S_1_, -S_2_, -D, and -R, with various sizes and shapes for self-healing anticorrosion coatings and presented a dynamic protective coating system with a self-healing anticorrosion function. ZIF-7 nanoparticles release the active BI inhibitor onto metal surfaces as healing agent, leading to a new barrier once exposed to the acid environment around the scratch areas. As determined by electrochemical impedance spectroscopy (EIS) studies, the corrosion resistance of carbon steel in 0.1 M hydrochloric acid solution improved remarkably when compared to that of the control samples with the embedding and homogeneous distribution of the ZIF-7 nanoparticles in polymer coating system. During a simulated corrosion process by scanning electrochemical microscopy (SECM), the dynamic polymer system exhibited its self-healing anticorrosion property. A variation in the concentration of embedded ZIF-7 nanoparticles from 0.5 to 7 wt.% allowed for obtaining the optimum conditions for anticorrosion performance, which was found to be the coating containing 1.7 wt.% ZIF-7-S_1_. Thus, the research group showed that ZIF-7, based on dynamic protective polymer systems, interacts with and has the ability to adapt to the surrounding acid environment, thereby leading to a new healed barrier on metal surfaces with anticorrosion functions [126].

#### 4.2.4. Bioactive Polymer Nanocomposite

The creation of bioactive polymer nanocomposite coatings with improved surface protection and biocompatibility has gained importance and raised a lot of concerns for orthopedic implant applications. Because it closely resembles the inorganic makeup of natural bone, hydroxyapatite (HA) is one of the most promising ceramic biomaterials. It is used as a covering for metallic implants, a medication carrier, and a bone graft [127,128]. Also, nanoscale HA has the ability to interact easily with fibronectin and vitronectin, which serve as the basic ligands of integrins possessing significant roles in cell adhesion. Moreover, the substitution of elements such as Mg^2+^, Sr^2+^, and F^−^ ions has been carried out and thoroughly examined in order to improve the durability and anticorrosion capabilities of HA in physiological environments.

Amongst the various CPs, poly(3,4-ethylene dioxythiophene) (PEDOT) has attracted significant considerations in biomedical applications attributed to the unique features and doping chemistry [129]. Because of its poor biocompatibility and unrestricted corrosion protection capabilities in physiological environments, PEDOT is not widely used as an implant coating material. Numerous studies have been conducted and various methods have been developed to create PEDOT and inorganic-based material composites in an effort to address this problem.

A unique poly (3,4-ethylenedioxythiophene) (PEDOT)-based nanocomposite coating with varying fluoro hydroxyapatite (FHA) nanoparticle concentrations was electrochemically synthesized by Kumar et al. on a recently discovered Ti-Nb-Zr (TNZ) alloy; this is a viable method to improve the surface features of TNZ implants. The FTIR, XRD, and Raman analyses demonstrated the successful synthesis of the PEDOT/FHA nanocomposite, while the XPS examination confirmed the chemical interaction between the FHA and PEDOT matrix. The homogeneous distribution of spherical FHA nanoparticles within the PEDOT matrix was demonstrated by SEM and TEM analysis. Studies using both Gram-positive and Gram-negative bacteria revealed that the coated TNZ substrates performed better against bacteria. Therefore, the study demonstrated that the PEDOT/FHA nanocomposite was one of the most effective and viable coatings for orthopedic implants [130].

#### 4.2.5. Epoxy-Based Nanocomposite

In the recent scenario, hetero-atom [131] doping provides an efficient way to inflect the properties of conducting polymers (CPs) for their engineering applications. Taking this lead into consideration, the limitations associated with CPs have to be eradicated for the enhancement of their properties. Rawat et al., in their work, proposed the introduction of boron in CPs as a dopant. The work was envisioned to perform the green synthesis of a novel nano-conducting polymer, nano poly(borophenyldiammine) PBPDA, via an easy and scalable approach. A novel route free from template surfactant and with no VOCs was utilized to form well-defined and highly conducting nanostructures. The fabrication process was easily performed, which did not require expensive reagents, conveniently in a green solvent of ethylene glycol and methanol. Also, the formulation of epoxy dispersed nanocomposite was accomplished using DMSO as a green solvent, an alternative non-toxic solvent, reducing VOCs and PBPDA used as a non-toxic anti-corrosive pigment in coating materials possessing anti-corrosion properties. The synthesized hybrid nanocomposite coatings displayed enhanced anticorrosive performance, which can be attributed to the presence of homogenously dispersed PBPDA nanoparticles in the epoxy matrix and the B moiety present increasing the conductivity of the nano PDA filler by 10^3^ times, acting not only as a strong barrier, inhibiting the penetration of corrosive ions at coating metal interface, but also contributing to protection through the redox mechanism (due to higher positive potential). Also, the strong adhesion between the coating material, (process mechanism and hydrogen bonding between PBPDA and epoxy matrix) and the metal surface serves as one of the chief reasons for the commendable corrosion resistance performance in NaCl medium [132].

Pourhashem et al. investigated the effects of visible-light-driven titanium dioxide (TiO_2_) nanotube/graphitic carbon nitride (g-C_3_N_4_) hybrids on the corrosion protection performance of epoxy coatings both in the dark and under visible-light irradiation. They carried out the synthesis of the TiO_2_ nanotube/g-C_3_N_4_ hybrids containing g-C_3_N_4_ in various amounts and silane-functionalized hybrids, followed by their characterization using XRD, TEM, SEM/EDS mapping, XPS, and N_2_ adsorption/desorption isotherms. The synthesized samples were then studied for their optical properties and visible-light photocatalytic performance. The photopotential measurements revealed that the epoxy coatings containing hybrids show more negative potential under visible-light irradiation because of the excitation of the electron–hole pairs and migration of the photogenerated electrons from the hybrids to the metal surface. However, as per the EIS results, the corrosion protection performance of the nanocomposite coatings decreases under visible-light irradiation. It also indicated that the addition of H30% and F-H30% effectively escalates the Z modulus of the epoxy composite coatings and also increases the service life of the coating systems in the dark, and E/0.3F was proved to provide the highest corrosion resistance, attributed to the concomitant effects of high surface area and band-gap structure; however, a decrease in the photocatalytic activity was observed after the silane functionalization of H30%. Their corrosion resistance was investigated by electrochemical impedance spectroscopy in NaCl solution, where it was shown that the epoxy coatings containing 0.3 wt.% hybrids have higher corrosion resistance when compared to that of the neat coating. Thus, the work showed that though nanomaterials have the affinity of increasing the corrosion protection performance of the polymer coatings, they have limited applications under light irradiation because the photocatalytic nanomaterials can accelerate the degradation of polymer coatings under light irradiation and increase the corrosion of coated steel substrates. While these nanomaterials can increase the corrosion resistance of polymer coatings in the dark, they have limited applications under light irradiation because the photocatalytic nanomaterials can accelerate the degradation of polymer coatings under light irradiation and increase the corrosion of coated steel substrates [133].

In 2022, the fabrication of a novel multilayer polymeric epoxy coating comprising of 3-(2-aminoethylamino) propyl dimethoxy methylsilane (AEAPS), Mo_2_C, and GO was carried out. Techniques such as EIS, polarization, and SECM studies were used to assess the protective act of epoxy-GO/AEAPS-Mo_2_C coating on mild steel in 3.5% NaCl solution. The highest coating resistance and the least aggregation of the coating was found when the optimum percentage of graphene oxide embedded Mo_2_C nanoparticles in the epoxy matrix was 2.0 wt.%. The coating resistance of EP-GO/AEAPS-Mo_2_C nanocomposite (7301.75 kΩ·cm^2^) shows its enhanced performance even after 360 h of immersion when compared to that of the plain epoxy (1.05 kΩ·cm^2^) coatings. The addition of GO wrapped molybdenum carbide in the epoxy matrix exhibited notable mechanical properties in terms of adhesion strength and hardness [134].

Rahmani et al. thermally devised an oxidized nanodiamond (OND) that was first modified non covalently with dodecylamine (DDA) as corrosion inhibitor. FTIR and TGA analysis revealed that the OND nanoparticle was effectively functionalized by DDA up to approximately 5 wt.% grafting content. Both OND and DND were loaded in epoxy (EP)/polyamine hardener matrix at the same concentration of 1 wt.% and applied on mild steel substrate. Investigation of the morphology of EP-DND and EP-OND by FE-SEM indicated good nano-dispersion of DND in the EP matrix. EIS analysis displayed that the low frequency impedance of EP-DND after a time duration of 30 days immersion in a 3.5 wt.% NaCl solution was found to be at least one order of magnitude higher than EP-OND and pure epoxy. Such excellent corrosion protection behavior was attributed to the effective barrier properties of EP-DND against corrosive species caused by the good dispersion of DND and efficient interaction within the epoxy matrix as well as the pH-responsive release of DDA as a corrosion inhibitor from the nanodiamond surface. A pull-off adhesion test revealed that the addition of ND (particularly DND) into the polymeric matrix strengthens the coating–substrate interaction. A superior anticorrosion performance was observed for EP-DND, attributed to the enhanced barrier properties and hydrophobicity of the coating (causing less corrosive species diffusion) [135].

Bahremand et al. demonstrated the coating of a steel surface with an eco-friendly Sm-based CC followed by its post-modification using a poly-dopamine-based biopolymer. The treated steel surface characterization was carried out using the SEM/EDS analysis and the electrochemical impedance spectroscopy (EIS) techniques. Also, the epoxy (EP) coating corrosion protection performance was investigated using the electrochemical impedance spectroscopy (EIS) analysis and salt spray test (SST). The EDS results displayed that a compact coating with high coverage was developed on the steel surface via Sm-PDA modification. In addition, SEM micrographs showed that the nano-and micro-roughness formation on the samarium and PDA treated samples, respectively, enhanced the EP adhesion to the steel surface through the mechanical interlocking mechanism. A graphene-like poly-dopamine film formation on the Sm-PDA treated specimen was confirmed by the Raman test results. When compared to the unmodified steel, all EP coated steel sheets provided better corrosion protection performance as affirmed by the EIS and SST results [136].

Pre-treatment of the CG sample having a thickness of about 25 nm through a facile technique of acid soaking followed by its acid treatment and then introducing it into the epoxy resin (EP) was found to escalate the toughness alongside keeping intact the high rigidity of the EP. The thickness was observed to decrease to about 1 nm, making the CG well dispersed in the epoxy matrix. The treated EP composite exhibited higher tensile strength and fracture energy. The EP composite displayed a higher glass transition temperature, making it suitable as cost-effective graphite nanoplates [137].

Dev et al. studied the fabrication of multiscale glass fiber/epoxy composites by invoking the graphene nanoparticles (GNPs) and zinc oxide nanoparticles (ZnO NPs), which showed a positive influence of NPs on the mechanical properties of composites. The compression molding technique was used to manufacture the composites with varying GNP contents, keeping the glass fibers and ZnO NPs content intact. The study outcomes display insights that promote the use of synthesized multiscale composites in the aerospace, automotive, and marine industries [138].

**Commercial Epoxy-based Nanocomposite**

The adhesion operating in between the nanoparticles and the epoxy matrix should be a proper one, as it displays a significant role in increasing the corrosion protection ability of the epoxy nanocomposites, thereby ensuring the uniform dispersion of nanoparticles within the resin, which affects the potential of the corrosion protection. Zhang et al., in their study, showed an improvement in the corrosion protection performance of C750 Gnp/epoxy nanocomposites attributed to the robust passive layer and surface barrier features by the composite coatings [139].

The hybrid coating with 90% acrylic resin blended with 10% epoxy resin displays better adhesion characteristics. The development of a hybrid acrylic polyol-epoxy polyol coating cured with aliphatic polyisocyanate exhibited corrosion protection when applied on mild steel panels. In order to escalate the adhesion of the hybrid polymer, polyisocyanate performed as a hardener, thereby escalating the hybrid polymer resin’s adhesion to the metal surface [140].

#### 4.2.6. Polymethyl Methacrylate (PMMA) Hybrid Nanoparticles

An efficient strategy to synthesize Hybrid nanoparticles (HNPs) with zinc oxide and polymethyl metha acrylate (inorganic/polymer) was carried out through the exploitation of the ultrasound approach. The fabricated HNPs were characterized employing transmission electron microscopy and X-ray diffraction. ZnO-PMMA-based HNPs provide significant protection properties to mild steel from corrosion when exposed to acidic condition. Electrochemical impendence spectroscopy (EIS) analysis was carried out to evaluate the corrosion inhibition performance of an MS panel coated with 2 wt.% or 4 wt.% of HNPs and its comparison with a bare panel as well as one loaded with only a standard epoxy coating. Tafel plot and Nyquist plot analysis interpreted that the corrosion current density (Icorr) decreases from 16.7 A/m^2^ for bare material to 0.103 A/m^2^ for 4% coating of HNPs. The developed coatings were tested for corrosion tests in salt solutions. The fabricated HNPs exhibited potential application in the corrosion inhibition of metal in an acidic environment. The metal life for a bare material was found to be 2–3 days, while it was 6 days for epoxy coating and increased up to 10 days for the MS panel coated with HNPs coating material [141].

Santos et al. used organic–inorganic hybrid coatings for the purpose of the corrosion protection of aluminum alloys, which proves as a promising alternative to the current methods based on chromate passivation. PMMA-silica hybrid coatings for thermal and anticorrosive applications were successfully fabricated by systematically varying the ratio of organic to inorganic phase as unsupported films and deposited onto an Al2024-T3 aluminum alloy. FTIR and NMR results revealed the bonding structure of the hybrids and the absence of low condensed silica species. The chemical and nanostructural properties of the hybrid coatings were evaluated employing the FTIR and small angle X-ray scattering (SAXS). Nanostructural analysis by SAXS revealed an escalated spacing between the nanosized silica nodes for higher polymer and silica content, resulting in larger silica domains. The low cost, versatile preparation method, small coating thickness, and cross-linked nanostructure provided by the environmentally compliant PMMA-silica coatings also displayed elevated thermal stability and efficient long-term corrosion protection against aggressive environments, thereby proving it to be a substitute for chromates in the corrosion protection of the aluminum alloy Al2024-T3 used in the aeronautic industry [142].

#### 4.2.7. Superhydrophobic Zinc Based Coating

In order to protect marine metals, superhydrophobic surfaces have proven to be an effective technology. A study proposed a facile method for fabricating a composite coating comprising of a zinc coating and superhydrophobic surface on a steel surface. The fabrication of the rough microstructure was carried out using sand particles. A low-surface-energy silicon nanofilaments layer (SNF layer) was grown in situ on the surfaces through alkaline catalysis of the geopolymer. The inherent alkaline species existing in the pore solution of the geopolymer triggered the hydrolysis-polymerization reaction of poly(methylhydrogen)siloxane (PMHS). After thermal curing, a superhydrophobic interface with a hierarchical micro/nanostructure can be assembled in situ on the coating surface. Characterization of the prepared samples was carried out using the SEM/EDS, XRD, CLSM, and XPS analysis methods. The water contact angle, the drag reduction measurement, and electrochemical analysis were used to test the superhydrophobicity performances. The prepared superhydrophobic zinc coating displayed a high water contact angle of 164◦ and showed a high drag reduction rate of 45% in water. The hydrophobicity of the coating was preserved in a 3.5% NaCl solution for a long duration of time. Due to the trapped air on the surface, the coating showed good corrosion protection for the steel substrate, and the cathode protection duration of zinc dust was prolonged. The designed steel coating displayed a 98% superhydrophobic repairable ability and excellent corrosion protection [143].

#### 4.2.8. Chitosan-Based Nanocomposite Coatings

Chitosan is a kind of N-deacetylated derivative of chitin, which is a natural polymer with inherent attractive properties such as low production cost, non-toxicity, biocompatibility, and pH sensitivity [144,145]. The differentiating characteristics of chitosan are attributed to its amine (–NH_2_) and hydroxyl (–OH) groups, which promote structural modification, aid the hydrolysis and dissolution of chitosan under acidic aqueous condition, and escalate the reaction of chitosan for providing homogeneous phases in chemical cross-linking networks. Also, chitosan displays an anti-corrosive nature because of the coordination of chitosan with the metal surface through –NH_2_ and –OH groups [146,147].

The hydrothermal synthesis of TiO_2_ nanotubes (TNT) in the anatase phase was executed by Pourhashem et al. and then chemically modified using aqueous chitosan solution (TCS). The synthesized TNT and TCS were characterized using XRD, XPS, and TEM analysis. Three varieties of epoxy composite coatings were fabricated as follows: (1) the epoxy composite coatings loaded with different wt.% (0.1, 0.5, and 1.0 wt.%) of TCS as nanofiller, (2) the epoxy coatings modified with 1 and 2.5 wt.% of aqueous chitosan solution as a polymer matrix modifier, and (3) the epoxy coatings modified with 2.5 wt.% of aqueous chitosan solution and loaded with different wt.% (0.1, 0.25, 0.5, and 1.0 wt.%) of TCS. The prepared composite coatings were evaluated for their corrosion protection performance by EIS and the salt spray test, while the adhesion strength of the coatings to the metallic substrates was measured by the pull-off adhesion test. As per the results, the characteristic properties of the composite coatings are greatly affected by the wt.% of nanofillers or the chitosan solution; meanwhile, the ECS-TCS0.5% coating (i.e., the epoxy matrix modified with 2.5 wt.% aqueous chitosan solution and loaded with 0.5 wt.% TCS nanofillers) displayed the highest protection against corrosion and the highest adhesion strength both in dry and wet pull-off tests compared to the other composite coatings. When both the polymer matrix is modified with the chitosan solution and the TNT is chemically modified with the chitosan, the chemical compatibility between the polymer matrix and the nanofillers enhances. Thus, a higher barrier effect comes into play using the chitosan acting as the bridge between the nanofiller and the epoxy matrix. It also passivates the metal surface, which leads to a decrease in the degradation of coating under a corrosive environment. So, the chitosan polymer acts as a non-toxic and natural polymer in anti-corrosion nanocomposite coatings through the ways of modifying the nanofillers and improvising the polymer matrix with chitosan polymer [148].

#### 4.2.9. Polyurethane-Based Nanocomposite

Owing to its inherent adaptability and desirable qualities since its origin, PU (polyurethane) is popular as a surface coating material. It has also emerged as a fascinating class of synthetic polymers that has the ability to achieve a unique molecular design tailored for a wide range of applications, including primers, adhesives, sealants, coatings, foams (flexible and rigid), elastomers, sports goods, medical devices, and others. Also, the distinguishing features of Pus, including the nature of the polyols and diisocyanates, the extent of crosslinking, and the curing conditions, contribute to the final properties of PUs. In addition to this, the proper fabrication of these materials effects the properties such as physicomechanical strength and surface hydrophobicity, which further extend their service life in the field of anticorrosive coatings [149].

Cardanol (Col) obtained from cashew nutshell liquid (CNSL), a meta-substituted phenol and agricultural byproduct of the cashew industry, has come up as a significant precursor for the fabrication of various polymers with specific applications amongst vegetable oils. The fabrication of renewable resource-based nanocomposite films/coatings (cardanol and Col) has evolved as a developing pathway, proving itself to be a sustainable and environment-friendly polymeric material attracting sincere attention. The synthesis of Co(II)- and Ni(II)-coordinated Col nanocomposites (Col-CPU/CoO and Col-CPU/NiO) was carried out by Kahn et al. through an easy, one-pot, environment-friendly, cost-effective, in situ approach. The use of these materials helped in the fabrication of mechanically robust, anti-corrosive, antibacterial, free-standing thin films and coatings found to be active against Gram-positive (*S. aureus* and *B. subtilis*) and Gram-negative (*E. coli* and *P. aeruginosa*) bacteria [150].

N. Arianpouya et al. investigated that the incorporation of polyurethane, zinc, and nano composite clay enhances the corrosion performance of zinc-rich coatings [151]. To study this effect, various percentages of montmorillonite clay nanolayers were incorporated into zinc-rich polyurethane nanocomposites. The data obtained from electrochemical tests indicated that the incorporation of clay nanolayers enhances the corrosion resistance of coatings, with the 2 weight% nanoclay with nanocomposite sample showing the best corrosion performance. In another study, N. Arianpouya et al. showed the synergistic effect of clay and zinc nanopigments. In the report, it was found that from EIS data, the inclusion of clay nanolayers boosts the corrosion protection of coating, with 2 weight% nanocomposite with nanoclay showing the best result [152].

#### 4.2.10. Nanostructured Carbon-Based Coatings

Pukha et al., in the paper titled “Corrosion-resistant nanostructured carbon-based coatings for applications in fuel cells based on bipolar plates”, describes the carbon coatings deposited by accelerated C60 ion beam irradiation onto the VT1-0 titanium alloy surface at varying substrate temperatures, seeing possible potential applications in fuel-cell bipolar plates. The temperature range for the fabrication of a conductive carbon nanocomposite coating (CNC) at various accelerating voltages (*Ua*) was also identified. The fabricated CNC comprises graphite nanocrystals embedded in an amorphous diamond-like matrix. Tribological studies showed the high wear resistance and high adhesion of CNC to titanium substrate, displaying potential for the stable operation of coated bipolar plates in mobile applications. CNC also helps in overcoming the limitation of corrosion-caused ICR increase, making the CNC-coated titanium a potential and promising candidate for substituting gold-coated stainless steel as a raw material for bipolar plates of proton-exchange membrane fuel cells [153].

#### 4.2.11. Nanostructured Al_2_O_3_-13TiO_2_ Coating for Corrosion Protection

Superhydrophobic coatings receive much attention for their widespread promising applications ranging from industry to our daily life. In the route of constructing superhydrophobic coatings, the introduction of nanoparticles into the polymer matrix plays a significant role. A durable coating having the potential of tackling the compatibility problem of inorganic and organic phases was developed by Zhu et al. formulated with hyperbranched polysiloxane modified silica nanoparticles and silicon-based acrylic polymers. These were found to be proficient in retaining superhydrophobicity even after being exposed to harsh conditions comprising immersion in acid–base solutions for a tenure of 12 days, immersion in boiling water for 3 hrs, and exposure to a high temperature of 150 °C for 24 h, as well as undergoing several mechanical damage tests such as 100 cycles of sandpaper abrasion, 40 cycles of tape peeling, and 200 g sand impact. The fabrication technology of superhydrophobic coatings finds unique advantages in climbable productivity, fewer equipment requirements, and minor process conditions, besides providing insights into the synthesis of superhydrophobic coatings with good compatibility of inorganic and organic materials [154].

The ultrasound-aided sealing with aluminum phosphate and silicone resin used to fill the pores in the subsonic plasma sprayed nanostructured Al_2_O_3_-13TiO_2_ coating improved the performance of the corrosion resistance of the coating. Also, as the ultrasonic environment enhanced the penetration of the sealant, the polarization resistance of the Al_2_O_3_-13TiO_2_ coating after being sealed with aluminum phosphate and with silicone resin was approximated to 6.3 times and 119.6 times greater than that of the as-sprayed Al_2_O_3_-13TiO_2_ coating, respectively. When compared with the aluminum phosphate sealant, the nanostructured Al_2_O_3_-13TiO_2_ coatings after ultrasound-assisted sealing with silicone resin influenced sophisticated corrosion resistance in 3.5 wt.% NaCl solution [155].

#### 4.2.12. Acrylic Nanocomposite

Acrylic-based coatings are popular for corrosion protection due to their high adhesion, clarity, and environmental resistance. However, adding nanoparticles may improve their barrier qualities, resulting in acrylic nanocomposites. Many researchers have combined acrylate coatings with various nanofillers to provide superior corrosion protection on metallic substrates. Proper integration allows for low-thickness coatings with high anticorrosive performance at a reasonable cost [156]. Ubong Eduok et al. found that zirconia-modified acrylic nanocomposite resins have been produced through integrating ZrO_2_NPs into varying grams of an acrylic base resin [157]. The inorganic/organic hybrid resin coating was applied to Q235 steel to resist corrosion in a 3.5 wt.% NaCl solution. The presence of ZrO_2_NPs in these acrylic coatings further inhibited natural channels through which corrosive ions and molecules may have infiltrated the coating, hence delaying steel corrosion. It was also observed that these particles improved the coatings’ bulk structural reinforcement and barrier performance.

Bozorg et al. investigated that the addition of nanosilica particles homogeneously dispersed into acrylic resin matrix showed the best corrosion protection, which was measured by the Nyquist plots as well as salt spray tests. The coatings’ performance was examined based on UV resistance, optical characteristics, and corrosion resistance [158]. The integration of inorganic nanophases in an acrylic polymer matrix has shown promise for creating high-performance hybrid coatings. A detailed study has been conducted, varying parameters such as precursor reagents, reaction time, temperature, solvent, and substrate type [156,159,160].

## 5. Future Perspectives

Nanostructured coatings display potential applications in variegated arenas, some of which are the engineering sectors, including the marine, space/aerospace, automotive, robotics, medicine (e.g., orthopedic, dental), sports, structure/architecture, defense, and energy systems fields. In order to explore the complete applicative potential of coatings, the major factors that are to be addressed include strategies for improving interfacial adhesion between coatings and substrates, the development of easy, scalable, and low-cost strategies for the preparation of the coating, and, also, the valuable insights from the natural systems for designing the coatings. The development of the mathematical models of the coatings would also provide a huge positive impact for designing the coating. This step would enable us to optimize the process of the coating and its properties before implementing it in real-world applications.

## 6. Conclusions

This review provides a comprehensive review on the corrosion protection behavior of nanostructured coatings and nanocomposite coatings. An extensive range of methods and materials have been studied in the field of corrosion protection coatings, thereby offering creative solutions. The development of coatings for corrosion applications is a stimulating endeavor given that variegated factors influence the properties and performance of coatings (coating material, composition, synthesis method, processing parameters, grain size, additives, reinforcements/fillers, operating environment, contact conditions, etc.). Several factors such as the selection of coating material/reinforcements, chemical complexity, and cost and time effectiveness in fabricating coatings make the development of effective coatings even more challenging. Developed nanostructured coatings and nanocomposite coatings have revealed excellent performance at a laboratory scale.

## Data Availability

No new data were created or analyzed in this study.

## Abbreviations

APS	Ammonium peroxydisulfate
AEAPS	3-(2-aminoethylamino) propyl dimethoxy methylsilane
BPA	Bisphenol A
BET	Brunauer–Emmett–Teller
BDDE	1,4-butanediol diglycidyl ether
CNO	Carbon nano-onion
CD	Cathodic disbondment test
Ce–La	Cerium–lanthanum conversion coating
CPs	Conducting polymers
CSA	Camphorsulfonic acid
CS	Chitosan
CEPD	Cathodic electrophoretic deposition
CLSM	Confocal laser scanning microscope
TCS	Chitosan modified TiO_2_ nanotubes
CNSL	Cashew nutshell liquid
CNC	Conductive carbon nanocomposite coating
CPCC	Chromate/phosphate conversion coated
DBSA	Dodecylbenzene sulfonic acid
DDA	Dodecylamine
DMSO	Dimethyl sulfoxide
DSC	Differential scanning calorimetry
DND	Dodecyl amine modified OND
EIS	Electrochemical impedance spectroscopy
EPA	Epoxy blend with polyaniline
EPM	Epoxy blend with polyaniline/MMT
EDS	Energy dispersive X-ray spectroscopy
EPE	Epoxy ester
EPZ	Epoxy/PANI-ZnO hybrid nanocomposite
epH	Epoxy-phenolic
FESEM	Field emission scanning electron microscopy
FTIR	Fourier transform infrared
FHA	Fluoro hydroxyapatite
GO	Graphene oxide
GPC	Gel permeation chromatography
PGHEP	Hydroxyl epoxy phosphate monomer
HA	Hydroxyapatite
HBP	Hyperbranched polyester-amide polymer
HNPs	Hybrid nanoparticles
HRTEM	High-resolution transmission electron microscopy
ICP	Intrinsically conducting polymers
ICP-OES	Inductively coupled plasma optical emission spectroscopy
ICR	Interface contact resistance
IPNs	Interpenetrating polymer networks
FT-IR	Infrared spectroscopy
LDH	Layered double hydroxides
MMT	Montmorillonite
MG	Modified GO
MNC	Modified NC
MIO	Micaceous iron oxide
MBI	2-mercaptobenzimidazole
KH-570	Methacryloxy propyl trimethoxyl silane
MSNs	Mesoporous silica nanoparticles
MF	Melamine formaldehyde
MD	Molecular dynamics
MC	Monte Carlo
MCPC	Melamine-cured polyester coating
NPs	Nanocomposite
NC	Nanoclay
OCP	Open circuit potential
OMMT	Organo-modified montmorillonite
OND	Oxidized nanodiamond
PEDOT	poly (3,4-ethylenedioxythiophene)
PANI	Polyaniline
PMHS	poly(methylhydrogen)siloxane
Pani-LGS	Polyaniline lignosulfonate-doped polyaniline
PCAIPs	Percent and coated alumina inhibitor particles
PMMA	Poly methyl methacrylate
PU	Polyurethane
PAniC	Polyaniline clay
PACN	Polyaniline clay nanocomposite
P_EF_	Protection efficiency
PPy	Polypyrrole
PDAP	PPy-deposited alumina particles
Ppy–MMT	Polypyrrole-montmorillonite
PCC	PPy/chitosan composites
PVA	Poly(vinyl) alcohol
PDA	poly-dopamine
PDMS	Poly(dimethylsiloxane)
PTh	polythiophene
PS	Polystyrene
PI	Polyimide
PBI	polybenzimidazole
PDP	potentiodynamic polarization
PEDOT	poly(3,4-ethylene dioxythiophene)
QM	Quantum mechanical
RM	rheometric mechanical spectroscopy
rGO	reduced graphene oxide
SST	Salt spray test
SAXS	Small angle X-ray scattering
SNF	Silicon nanofilaments
SD	Sodium dodecyl sulfate
SPANi	Sulfonated polyaniline
SEM	Scanning electron microscope
TGA	Thermogravimetric analysis
TA	Tannic acid
TNZ	Ti-Nb-Zr
TEM	Transmission electron microscopy
TNT	TiO_2_ nanotubes
TG/DTA	Thermogravimetry differential thermal analysis
UV–Vis	UV–vis absorption spectra
UF	Urea formaldehyde
VOCs	Volatile organic compounds
WAV	Water-based alkyd varnish
WTM	Wet transfer method
WVT	Water vapor transmission
WPU	Waterborne polyurethanes
XPS	X-ray photoelectron spectroscopy
XRD	X-ray diffraction
ZRP	Zinc-rich epoxy primer’s
ZRP	Zinc-rich paints
ZAPP	Zinc aluminum polyphosphate
ZIF	Zeolitic imidazole framework
